# Fundamentals and Advances in Programmable Peptide Hydrogels for Multifunctional Biomedical Applications: A Review

**DOI:** 10.3390/gels12060527

**Published:** 2026-06-11

**Authors:** Yihao Zhao, Zhe Zhang, Mingyang Jiang, Cancan Xu, Zhiwei Shen

**Affiliations:** 1 Queen Mary School, Jiangxi Medical College, Nanchang University, 999 Xuefu Road, Nanchang 330031, China; zhaoyihao0409@163.com (Y.Z.); zhangzhe083102@163.com (Z.Z.); 2 Jiangxi Province Key Laboratory of Bioengineering Drugs, School of Pharmacy, Jiangxi Medical College, Nanchang University, Nanchang 330031, China; jiangmingyang0127@163.com (M.J.); 15979909748@163.com (C.X.); 3 National Engineering Research Center for Bioengineering Drugs and Technologies, Institute of Translational Medicine, Nanchang University, Nanchang 330031, China

**Keywords:** peptide hydrogels, peptide self-assembly, programmable biomaterials, stimulus-responsive hydrogel, tissue engineering, drug delivery, immunomodulation, biofunctionalization, regenerative medicine

## Abstract

Programmable peptide hydrogels represent advanced supramolecular biomaterials featured with customizable molecular sequences and tunable self-assembly behaviors, which can biomimetically reconstruct the structural and microenvironmental complexity of native extracellular matrix. This review systematically elaborates the molecular engineering advances of programmable peptide hydrogels following a hierarchical logic from fundamental mechanisms to translational applications. We first interpret the intrinsic self-assembly mechanisms driven by non-covalent interactions and the regulatory effects of typical external microenvironmental stimuli. On this basis, we summarize core rational design principles, covering stimuli-responsive structural optimization, biofunctional modification, and the tunable regulation of physical properties, degradability and immunogenicity. Furthermore, we correlate multi-scale structural features (nanostructures, porous architecture and mechanical properties) with their versatile biomedical functions, and comprehensively discuss their cutting-edge applications in tissue regeneration, targeted drug and gene delivery, cell-mediated therapy, immunomodulation, and anti-infective treatment. Finally, we identify critical translational barriers including batch-to-batch inconsistency, immunogenic risks, and in vivo performance instability, and highlight future directions involving multi-stimuli-responsive systems, artificial intelligence-assisted design, computational modeling, and hybrid material construction. This work systematically clarifies the structure–property–function relationship of peptide hydrogels, and underscores their great potential as next-generation platforms for precision regenerative medicine and targeted disease intervention.

## 1. Introduction

In recent years, the development of regenerative medicine and tissue engineering has put precise tissue repair and functional regeneration at the core of modern biomaterials research. With their flexible molecular design, excellent biocompatibility, and ability to form well-defined three-dimensional structures, such systems can significantly improve local therapeutic effects and reduce systemic adverse reactions, closely mimicking the complex microenvironment of natural extracellular matrix (ECM). Therefore, peptide hydrogels have become an important research hotspot at present [1]. By reasonably designing amino acid sequences and accurately regulating the self-assembly process, these hydrogels can form a stable 3D scaffold to support cell proliferation and regulate cell adhesion, migration and differentiation through specific biochemical signals to effectively improve the effect of tissue regeneration [2].

Enriching the function of peptide hydrogels can significantly expand their application in complex tissue repair. For example, in the repair of osteochondral defects, layered hydrogels can be constructed to release specific biochemical signals from each layer. These signals can synchronously regulate chondrogenesis and osteogenesis, thereby achieving spatial coordination and integration between cartilage and bone tissues [3,4]. In the study of central nervous system (CNS) repair, targeted peptide motifs (such as simulating sequences of N-calcium mycoprotein) are thought to help regulate cell adhesion and interfacial interactions. However, the existing research mainly focusses on the vascular damage repair model, and the direct experimental evidence for promoting CNS axon extension and synaptic formation is still limited [5].

In addition to playing a role in structural reconstruction, peptide hydrogels have shown great potential in many application fields such as cell therapy, immunoregulation and targeted drug delivery [6,7]. By integrating anti-inflammatory drugs and embedding immunomodulatory peptides, such systems can downregulate the expression of pro-inflammatory cytokines, inhibit excessive immune activation, and promote tissue repair in inflammatory or damaged microenvironments [8]. Moreover, the inherent adjustability and stimuli-responsive characteristics of peptide hydrogels enable them to respond to specific pathological signals and release therapeutic drugs on demand [9]. This function is highly advantageous in complex clinical situations, such as chronic inflammation, intractable wounds and tumor treatment, which can effectively improve local therapeutic effects and reduce systemic adverse reactions [10]. Furthermore, by introducing antimicrobial peptides (AMPs) domains with membrane-destructive activity, biological materials can be given broad-spectrum antibacterial ability and have a low risk of drug resistance induction [11,12,13,14].

Although peptide hydrogels have shown good prospects in preclinical research, their clinical translation is still hindered by many limiting factors, especially insufficient structural stability and differences between production batches [15]. In addition, the patient’s specific parameters (including local pH, enzyme environment and immune ability), coupled with tissue-dependent degradation dynamics, will affect the therapeutic efficacy [16]. In response to the above problems, existing research is improving the repeatability, mechanical stability and long-term biosafety of materials in clinical applications by optimizing peptide sequences, reducing immunogenicity, and constructing reversible functionalization strategies [17].

Against the above research background and existing challenges, this review systematically summarizes the fundamental mechanisms, rational design strategies, and multifunctional biomedical applications of programmable peptide hydrogels. Specifically, this paper first elaborates the core non-covalent driving forces dominating peptide self-assembly, including hydrogen bonding, hydrophobic interactions, π–π stacking, and electrostatic forces, and discusses the dynamic hierarchical pathways and structural evolution characteristics of peptide assembly. Subsequently, it comprehensively clarifies the intrinsic regulatory mechanisms of typical external stimuli (pH, temperature, light, and enzyme) on peptide self-assembly behavior, revealing the essential correlation between microenvironmental stimulation and hydrogel network formation. On the basis of these fundamental theories, this review further focuses on the modular design principles of intelligent peptide hydrogels, covering single- and multi-stimuli-responsive structural optimization, diversified biofunctionalization strategies for cell adhesion, antibacterial and immunomodulatory modification, as well as the precise regulation of macroscopic physical properties, including mechanical stiffness, porosity, degradability, and immunogenicity. Furthermore, the representative biomedical advances of programmable peptide hydrogels in tissue regeneration, targeted drug delivery, and precise disease intervention are systematically summarized. Finally, we highlight the current bottlenecks restricting their clinical translation and prospect future development trends of next-generation intelligent peptide biomaterials. This hierarchical and structured overview aims to provide a comprehensive and logical theoretical reference for the rational design, performance optimization, and clinical transformation of multifunctional programmable peptide hydrogels.

## 2. Self-Assembly Mechanism

**Figure 1:**
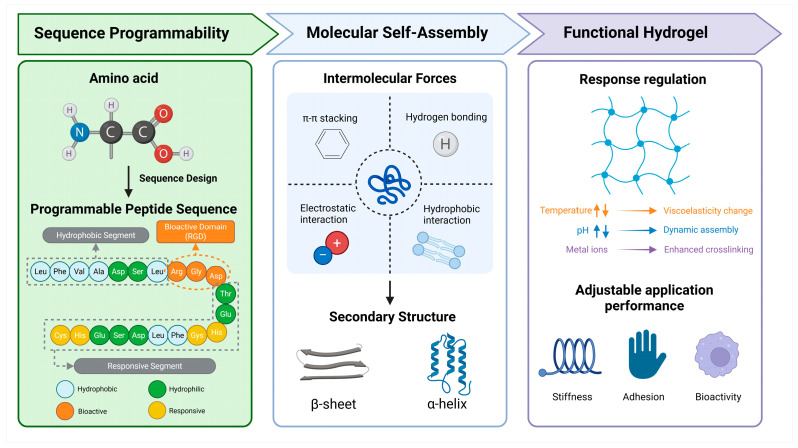
Design strategy and functional transformation diagram of programmable peptide hydrogels. The peptide sequence that is rationally designable is composed of amino acids, including hydrophobic, hydrophilic and biologically active fragments. Driven by hydrogen bonds, electrostatic action and hydrophobic interactions, polypeptides self-assemble to form regular secondary structures (β sheets or α helices), and finally form a supramolecular network, forming a responsive hydrogel with mechanical, adhesion and biological activity. Created in BioRender. Zhao, Y. (2026), https://BioRender.com/mbyuune (accessed on 22 April 2026).

The formation of peptide hydrogels is essentially an accurate and adjustable molecular self-assembly process. In aqueous environments, peptide monomers are arranged in an orderly manner through a variety of non-covalent forces (including hydrogen bonds, hydrophobic interactions, electrostatic interactions and π–π stacking), gradually undergo specific conformational transformations, and finally construct a three-dimensional hydrogel network structure [18,19]. Unlike traditional hydrogels that mainly rely on chemical crosslinking, the formation of peptide hydrogels does not require complex chemical modification. Relying on the designability of the peptide sequence itself and the responsiveness to the environment, self-assembly can be realized (Figure 1) [6,7].

The controllability of peptide self-assembly comes from its multi-level aggregation path and high responsiveness to external stimuli [20]. By regulating pH, temperature, and ionic strength or adding specific enzyme triggers, the secondary structure and aggregation state of the peptide chain can be accurately controlled, thus directly changing the gelation rate, network density, mechanical stiffness and biocompatibility of the hydrogel [21]. In tissue engineering, this regulation is very important: the cell microenvironment should not only provide stable mechanical support, but also be dynamic to adapt to cell adhesion, migration, proliferation and differentiation [22].

Peptide hydrogels are sensitive to microenvironmental changes and are rationally designable for structural dynamics. They have outstanding advantages in the preparation of tissue supports with strong adaptability and are suitable for the physiological environment [12]. This inherent flexibility helps to improve the compatibility and functional integration between materials and tissues, thereby enhancing their application potential in the fields of regenerative medicine and tissue reconstruction [23].

### 2.1. Intermolecular Forces

Recent studies have shown that the self-assembly of peptide hydrogels is driven by the fine synergy of multiple non-covalent forces at the molecular level. These forces jointly regulate the assembly rate and path, and also directly determine the mechanical stiffness, structural stability and functional characteristics of the supramolecular network [18]. The synergy of these interactions endows the hydrogel with both directional specificity and thermodynamic stability. These properties confer unique physicochemical characteristics on the hydrogel, enabling its broad application in the biomedical field.

In a variety of non-covalent forces, hydrogen bonds are the core stabilizing factors and play a key role in maintaining the integrity of the three-dimensional skeleton of peptide hydrogels [19,24]. The amide bonds of the peptide main chain and the hydroxyl group, amide group and other polar side chains together form a regular and stable hydrogen bond network, guiding the peptide chain to form a specific secondary structure [25,26]. Under this structure, β sheet forms a plane structure, so that nanofibers are arranged and stacked horizontally; α helix has a rod-shaped configuration with good elasticity, so that the hydrogel can still maintain the mechanical stiffness during deformation [24,27]. These secondary structures are stable and arranged, providing a spatial skeleton for the subsequent assembly of more complex advanced supramolecular networks [28].

The hydrophobic effect is the main thermodynamic driving force for the self-assembly of polypeptides and the formation of hydrogels, while hydrogen bonds play a secondary but key role in stabilizing the secondary structure [29]. Polypeptide fragments containing phenylalanine, leucine, valine and other non-polar residues will be phase-separated in aqueous environments and spontaneously aggregate to form dense hydrophobic clusters [30]. This self-aggregation reduces the free energy of the Gibbs system, transforms the disordered peptide chain into a tightly arranged supramolecular structure, and then activates the nucleation of fibrous nanostructures [29]. Notably, the stability and aggregation degree of hydrophobic microdomains directly affect the gelation rate, and determine the density and mechanical toughness of the hydrogel network [31,32].

In addition to hydrophobic aggregation, the π–π stacking between aromatic residues further stabilizes the core structure of nanofibers [33]. The side chains of phenylalanine, tyrosine and tryptophan can be arranged face-to-face or staggered in parallel to form a hydrophobic sandwich and enhance the internal binding force of the fiber [34]. This orderly aromatic accumulation not only improves the mechanical stiffness of the hydrogel matrix and enhances its shear resistance [24], but in some functional structures it also determines the spatial distribution and utilization of bioactive sites. The regulation of aromatic ring orientation will directly affect the identification of the cells and matrix in the scaffold and the subsequent biological effect [35].

Electrostatic action is the key regulatory factor of peptide hydrogel assembly, which directly affects the fiber aggregation rate and spatial arrangement [36]. The balance of gravity and repulsive force between charged residues directly determines the speed of structural assembly. Under low ionic intensity, the electrostatic rejection of the same side chain will weaken the fiber aggregation and delay the formation of gel [37]. On the contrary, adding an appropriate amount of salt can produce charge shielding, weaken the repulsive force, and make the main chain of the peptide chain more densely stacked to form a stable supramolecular structure [38,39]. pH also plays a key role: in an acidic environment, the protonation of negative groups can reduce electrostatic rejection and promote self-assembly; in an alkaline environment, deprotonation will produce more negative charges and destroy the stability of the gel network [40]. For polypeptides rich in cationic residues, the effect is the opposite: deprotonation under alkaline conditions can weaken intra-chain rejection, enhance aggregation, and make it easier to form hydrogels.

These intermolecular forces work together to construct a dynamic hierarchical structure in the process of self-assembly. Hydrogen bonds guide the formation of hydrophobic cores, hydrophobic aggregation enhances the stability of aromatic ring accumulation, and the electrostatic action is dynamically regulated: it affects the aggregation speed in the early stage and maintains the structural regularity in the later stage [19]. This multi-dimensional synergy makes the structure and function of peptide hydrogels highly adjustable, laying a reliable molecular foundation for its extensive application in the field of biomedicine [6,7].

### 2.2. Dynamic Self-Assembly Pathways

Driven by the synergistic non-covalent intermolecular forces summarized above, peptide monomers proceed through thermodynamically favorable nucleation and elongation cascades, enabling the stepwise formation of sophisticated hierarchical nanostructures via distinct self-assembly pathways. At the macroscopic level, peptide self-assembly proceeds through a cascade of discrete yet interconnected phases that align well with classical nucleation and growth theory. Initially, peptide monomers in aqueous solution predominantly exist in a dynamic equilibrium between random-coil conformations and partially stabilized secondary structural motifs such as β-turns or α-helical segments [40,41]. During this pre-nucleation stage, molecular coordination is largely diffuse, with only transient hydrogen bonds and short-lived hydrophobic clusters forming sporadically among individual chains [41]. As non-covalent forces progressively intensify, peptides undergo a thermodynamically driven reorganization characterized by hydrogen bonding, hydrophobic collapse, electrostatic complementarity, and π–π stacking. This cooperative interplay enables stable proto-oligomeric assemblies that function as critical nuclei, in accordance with the nucleation–elongation model of supramolecular polymerization [42]. Subsequently, these nuclei act as structural templates that direct the anisotropic growth of ordered, higher-order fibrillar or lamellar architectures, ultimately giving rise to the mature hydrogel network [42,43].

Once favorable thermodynamic and kinetic parameters are met, the preformed oligomers undergo directional elongation, giving rise to either one-dimensional nanofibers or two-dimensional, sheet-like assemblies [42]. High-aspect-ratio nanofiber is the main bearing structure to ensure the structural integrity and mechanical stiffness of the hydrogel matrix; the two-dimensional sheet structures form a dense surface area to improve the barrier and retention capacity [43]. These fibers and the sheet structures are entangled with each other in the three-dimensional skeleton to form a continuous molecular network, which can entrap large amounts of water. This water-containing reticular structure eventually forms a macroscopically uniform hydrogel whole.

Among the many modes of peptide self-assembly, the assembly path mediated by the β sheet is still the most thorough one. Under this mechanism, the single polypeptide chain adopts a parallel or anti-parallel arrangement, and the dense arrangement structure formed by intermolecular hydrogen bonds further stabilizes its spatial orientation, thus forming a regular layered structure [44]. The ordered sheet structures extend in a single direction, forming a stable fiber skeleton with a high aspect ratio [43]. These slender fibers are intertwined with each other in three-dimensional space, eventually forming a dense structurally and mechanically stable hydrogel network [45].

Another typical assembly path is the transition of polypeptides from α helix to β sheet secondary structure. Under environmental stimulation such as pH shift, temperature increase or ionic strength change, this conformation rearrangement can occur in specific peptide sequences [40]. Polypeptides change from α helix to β sheet, which can significantly accelerate nucleation and fiber extension, and then quickly build a three-dimensional supramolecular network [40]. This rapid gel characteristic is of great application value in biomedical scenarios that require immediate mechanical fixation, such as hemostasis and emergency tissue reinforcement [45,46].

In addition to such well-established assembly pathways, some artificial transformation and natural peptides can also realize self-assembly through coiled coils or radial stacking to form hollow nanotubes, spherical aggregates and other characteristic nanostructures [47]. Such a supramolecular structure has an internal cavity, which can contain drugs, nucleic acids or biologically active molecules to improve the delivery targeting and avoid the early degradation of the contained substances [48]. Therefore, peptide hydrogels are applicable not only as a mechanical support scaffold, but also as a stimulus-responsive carrier to realize the local and controlled release of drugs [9].

It should be emphasized that the self-assembly path of peptides is not only determined by the amino acid sequence. The assembly results are mainly regulated by the dynamic equilibrium of thermodynamic driving force and kinetic limitations, and are also affected by external environmental factors such as temperature, pH, ionic strength and enzyme activity [40]. Therefore, the rational design of functional peptide hydrogels needs to combine molecular construction with environmental regulation to achieve the optimal balance of mechanical stiffness, biological function and clinical application through the coordinated matching of structure and environmental parameters [49].

### 2.3. External Stimuli-Modulated Self-Assembly Mechanisms

The spontaneous assembly of peptide hydrogels is fundamentally governed by amino acid sequences, while exogenous stimuli enable precise spatiotemporal modulation of self-assembly behaviors via regulating intermolecular interactions and molecular configurations [20]. Multiple external physical and chemical triggers can precisely intervene in the nucleation, fiber extension and crosslinking processes of peptide molecules, thereby altering the microscopic network structure and mechanical properties of hydrogels. This section focuses on the basic regulatory mechanisms of typical external stimuli on peptide self-assembly, including pH, temperature, light and enzyme stimulation, which lays a theoretical foundation for the functional design of intelligent hydrogels [50].

pH stimulation regulates peptide hydrogel assembly mainly by changing the protonation state of ionizable amino acid side chains, which further balances intermolecular hydrogen bonding and electrostatic interactions [51]. Under acidic conditions, acidic amino acid residues (glutamic acid, aspartic acid) undergo protonation modification. This process weakens the original electrostatic interaction between positive and negative charges and the repulsion between homogeneous charges among peptide molecules [52,53], inhibits the aggregation and growth of nanofibers, and ultimately forms a loose hydrogel network structure with low crosslinking density [54]. In contrast, under near-physiological neutral pH conditions, the protonation state of peptide side chains is stable, hydrogen bonding and hydrophobic interactions dominate the self-assembly process, and peptide fibers extend sufficiently and crosslink densely, constructing a compact network with enhanced structural stability and mechanical strength [55].

Temperature is a critical physical factor affecting the thermodynamic behavior of peptide self-assembly. Temperature fluctuation changes the free energy distribution of hydrogen bond binding enthalpy and hydrophobic interaction strength in peptide systems [56]. At low temperature or room temperature, the hydrophobic segments of most intelligent peptides are fully hydrated, and peptides exist in a uniformly dispersed sol state with weak intermolecular interactions. With the increase in temperature, the hydration layer on the surface of hydrophobic amino acid segments is destroyed, and the hydrophobic aggregation effect is significantly enhanced. This thermodynamic change drives rapid nucleation and crosslinking of peptide molecules, promotes the formation of three-dimensional nanofiber networks, and completes the sol–gel phase transition. The hydrophilic–hydrophobic ratio of peptide sequences determines the temperature sensitivity threshold and gelation rate of hydrogels [12].

Light-responsive assembly relies on photochemical conformational transformation of functionalized peptide molecules. Photosensitive groups such as azobenzene and coumarin are covalently modified on peptide backbones and can produce specific photoisomerization or crosslinking reactions under irradiation of specific wavelength light [57]. The photostimulation-induced cis–trans isomerization of photosensitive groups directly changes the spatial conformation of peptide chains, disrupts or reconstructs intermolecular stacking modes, and thus realizes the reversible switch of peptide self-assembly and depolymerization [9]. Adjusting the light wavelength, irradiation time and irradiation area can precisely control the molecular arrangement and network formation degree of peptide hydrogels at the microscopic level [58].

Enzyme-responsive assembly is a biological microenvironment-triggered self-assembly mode dependent on specific enzymatic cleavage reactions. Functional peptide sequences are usually modified with protective fragments to shield self-assembly active sites, making peptides stably dispersed in solution [59]. In specific pathological microenvironments rich in matrix metalloproteinases (MMP-2, MMP-9, etc.), the protective fragments on peptide chains are specifically cleaved and removed by corresponding enzymes. The exposed active assembly sites initiate peptide nucleation and fiber aggregation, and finally form stable local hydrogel networks. The whole assembly process is strictly dependent on the specific recognition and cleavage of enzymes, showing high biological specificity [60].

In summary, different external stimuli regulate peptide hydrogel assembly through distinct basic mechanisms: pH modulates electrostatic interactions and hydrogen bonding balance via protonation and deprotonation, temperature controls hydrophobic aggregation and thermodynamic assembly behavior, light induces reversible conformational transformation of functional peptides, and enzyme stimulation triggers targeted assembly through specific biological cleavage. These multi-level regulatory mechanisms of molecular configuration, nucleation kinetics and aggregation paths jointly determine the gelation rate, network compactness and mechanical properties of peptide hydrogels [9,20].

## 3. Design Principles

As elaborated in the previous section, the spontaneous self-assembly of programmable peptide hydrogels is dynamically governed by intrinsic peptide sequences and exogenous microenvironmental stimuli, where non-covalent intermolecular forces and multi-path assembly behaviors endow these supramolecular materials with tunable nanostructures, mechanical properties, and environmental responsiveness. In-depth understanding of these fundamental assembly mechanisms and regulatory rules provides a solid theoretical basis for the targeted structural optimization and functional customization of peptide hydrogels. To further transform the inherent physicochemical responsiveness of peptide hydrogels into controllable biomedical functions and adapt to the complex and diverse requirements of regenerative medicine, targeted drug delivery, and disease microenvironment intervention, it is essential to establish systematic and standardized molecular design principles. On the basis of clarifying the correlation between peptide molecular structure, assembly behavior and material performance, rational structural modification and functional module integration can be implemented to precisely regulate the responsive characteristics, biological activity, physical properties and in vivo performance of hydrogels, thereby constructing high-performance programmable peptide hydrogel platforms adaptable to diverse biomedical scenarios. The following section systematically expounds the core design strategies of responsive regulation, biofunctionalization, physical performance tuning, and biosafety optimization of peptide hydrogels.

### 3.1. Responsive Design

Based on the basic stimulus-responsive assembly mechanisms of peptides clarified above, the core goal of hydrogel engineering design is to endow materials with intelligent adaptive functions, so as to meet the precise application requirements of biomedicine. By rationally embedding responsive functional units into peptide sequences, the controllable transformation of hydrogel microstructure and macroscopic functions can be realized by targeting physiological and pathological microenvironments, achieving high-precision targeted therapy, controlled drug release and in situ tissue repair [61,62]. This section focuses on the engineering design ideas, functional optimization strategies and application advantages of single and multi-responsive peptide hydrogels.

pH-responsive peptide hydrogels are the most maturely developed intelligent delivery system in responsive engineering design, which is widely used for targeted intervention of acidic pathological microenvironments such as tumor tissues and chronic inflammatory sites [63]. The core design strategy is to construct pH-sensitive peptide sequences rich in charged amino acids (glutamic acid, lysine, etc.). Utilizing the characteristic that pathological acidic microenvironments can change the ionization state of peptide side chains and reconstruct intermolecular force balance, the hydrogel is designed to realize in situ targeted assembly only in lesion areas [64]. This localized gelation design can precisely build drug storage reservoirs and cell scaffold structures at lesion sites, avoid abnormal assembly in normal physiological environments, effectively reduce toxic and side effects on surrounding healthy tissues, and significantly improve the safety and accuracy of tumor and inflammatory disease treatment [65].

Temperature-responsive peptide hydrogels are typical injectable intelligent materials, and their core engineering design focuses on the precise regulation of the peptide hydrophilic–hydrophobic segment ratio and thermal response threshold [50,66]. According to the thermodynamic assembly characteristics of temperature-sensitive peptides, researchers optimize sequence structure to make peptides maintain a low-viscosity sol state at room temperature, which is convenient for in vitro preparation, long-term storage and minimally invasive injection administration [9,67]. After being injected into the body and reaching the physiological temperature environment, the peptides rapidly undergo hydrophobic aggregation and sol–gel phase transition to form stable three-dimensional crosslinked nanofiber networks [68]. This in situ thermal gelation design can efficiently load and fix therapeutic drugs at target sites, maintain sustained high local drug concentration, avoid systemic diffusion of drugs, reduce adverse systemic reactions, and provide a reliable technical scheme for minimally invasive and precise targeted therapy.

**Figure 2:**
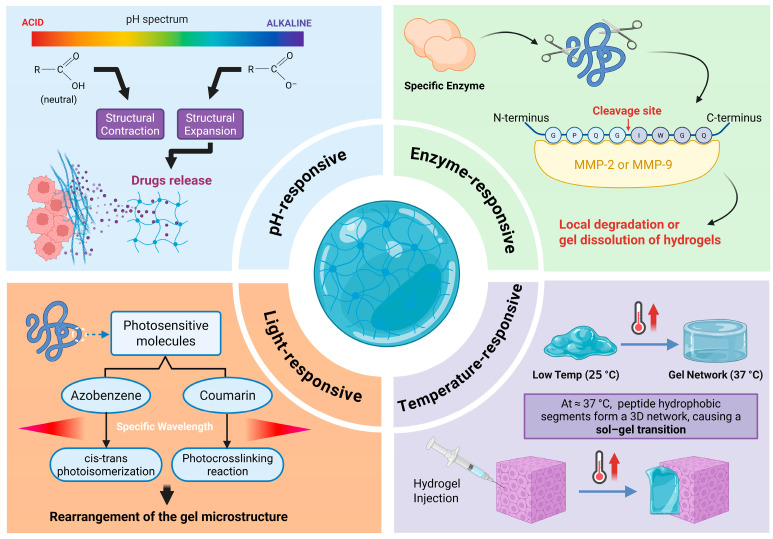
Responsive design mechanisms of programmable peptide hydrogels. The hydrogels respond to environmental signals including pH response (structural contraction or expansion to realize controlled drug release [76]), enzyme response (site-specific enzyme cutting, local degradation [77]), light response (photosensitive isomerization or photo-crosslinking [78]), and temperature response (sol–gel transformation occurs near physiological temperature [79]). Relying on these mechanisms, the structure, degradation rate and function of hydrogels can be accurately regulated to meet the needs of high-end biomedical applications. Created in BioRender. Zhao, Y. (2026), https://BioRender.com/mbyuune (accessed on 22 April 2026).

Light-responsive peptide hydrogels adopt a non-invasive optical control engineering strategy, realizing programmable and spatiotemporally precise biomedical applications. The key design is to covalently graft photosensitive groups such as azobenzene and coumarin derivatives onto peptide backbones to construct optically controllable peptide precursors [69]. Under specific wavelengths of light, these groups undergo cis–trans photoisomerization or light-driven crosslinking reactions, which alter the polypeptide conformation and rearrange the supramolecular structure [70]. Benefiting from the high spatial and temporal resolutions of optical stimulation, this design enables micron-level precise construction of cell culture scaffolds in vitro and on-demand local drug release in postoperative lesion sites in vivo, which can achieve accurate disease intervention while reducing secondary damage to normal tissues, and has great application potential in programmable cell culture and precise postoperative rehabilitation treatment [71]. Such a photocontrol strategy fully reflects the multifunctionality of responsive peptide hydrogels in programmable biomedical applications (Figure 2).

Multi-stimuli collaborative responsive design is the latest engineering development direction of intelligent peptide hydrogels, which aims to break through the limitation of the low targeting specificity of single-responsive systems. The core design idea is to integrate two or more independent responsive functional modules (pH-sensitive sequences, MMP enzyme-cleavable sites, temperature-sensitive units, etc.) on a single peptide skeleton to construct dual- and multi-responsive composite systems [72,73]. Taking pH–enzyme dual-responsive hydrogel as a typical example, the integrated responsive units can simultaneously identify the acidic microenvironment and high enzyme expression characteristics of tumor and inflammatory lesions, and only trigger structural transformation and targeted drug release under the co-stimulation of dual pathological signals [74,75]. This collaborative design strategy effectively avoids non-specific activation of hydrogels in normal tissues, significantly improves the targeting accuracy and biosafety of controlled drug release, and provides a more flexible and reliable material platform for the treatment of complex refractory diseases such as malignant tumors and infected wounds. To facilitate a systematic comparison of the diverse responsive design paradigms discussed above, the key strategies and representative features are concisely summarized in Table 1.

**Table 1:** Comparative summary of molecular design strategies for programmable peptide hydrogels.

Design Strategy	Representative Peptide Motif	Crosslinking Mechanism	Key Characteristics	Functional Applications	Representative References
Supramolecular Self-Assembly	MAX1; (FKFE)_2_, (FEFK)_2_	Physical: Hydrogen bonding, hydrophobic/π–π interactions, β sheet stacking; pH/ionic strength-triggered folding	High biocompatibility, injectability, ECM-mimetic nanofibrous network, tunable viscoelasticity, shear thinning	3D cell culture, soft tissue scaffolds, biosensors	[80]
Peptide-Polymer Conjugates	Peptide–PEG; peptide–PEG dual networks	Stereo-complexation; chemical crosslinking	Enhanced mechanical stiffness, dual-network toughness, controlled degradation, modular bioactivity	Load-bearing tissue repair, sustained large-molecule delivery	[12,17]
Stimuli-Responsive Design	HBD peptide; MMP-9 binding peptide	Redox/light; enzyme-cleavable; pH/temperature-triggered assembly; metal coordination	On-demand payload release, adaptive stiffness, ROS/enzyme sensitivity	Targeted drug delivery, diabetic wound healing, anti-inflammatory therapy	[81,82]
Multicomponent Co-Assembly	Glycyrrhizic acid (GA) + cryptotanshinone–peptide (CU); peptide + laponite (LAP) nanoparticles	Non-covalent co-assembly; photo-crosslinking of pre-assembled nanoparticles	Synergistic bioactivity, hierarchical nanostructure, improved stability	Infected wound therapy, corneal repair	[83,84]
Bioactive Motif Integration	RGD, QGT-IK gradient peptides	Enzymatic crosslinking; NHS–amine covalent bonding; heparin-mediated crosslinking	Tunable ligand density, integrin-specific adhesion, immunomodulation, hemostatic activity	Immune cell recruitment, non-compressible hemorrhage control, angiogenesis promotion	[46,85,86]

### 3.2. Biofunctionalization Strategies

Previously, peptide hydrogels were served merely as inert structural scaffolds. Currently, in order to actively guide cell behavior and regulate molecular processes in tissue repair, biofunctionalization has become a key strategy in the design of biological materials. Its core is to introduce biologically active sequences into the polypeptide chain so that they can specifically bind to cell surface receptors or ECM components [87]. This molecular modification combines the mechanics, structural characteristics and biological signaling of hydrogel to construct a dynamic guided microenvironment with self-regulation ability [88]. The functionalized peptide hydrogels, which have both structural support and biochemical interaction, provide a flexible and feasible platform for regulating the regeneration process of complex tissues.

The commonly used method of biofunctionalization is the introduction of short peptide fragments that can mediate cell recognition and adhesion. The Typical RGD (arginine–glycine–aspartic acid) sequence can bind to the bind integrin receptors with high affinity, effectively promoting the firm adhesion, full spread and directional migration of cells on the surface of hydrogels [89]. Likewise, the IKVAV (Ile–Lys–Val–Ala–Val) motif, originally identified within laminin, engages selectively with neural ECM constituents, facilitating neuronal lineage commitment and guiding neurite outgrowth [90]. If these biologically active epitopes are uniformly distributed at the nanometer level in the hydrogel surface and internal network, they can provide stable anchor points and clearly located signal centers for in situ cells and infiltrating cells in tissue regeneration [91]. This spatially controlled layout can significantly improve the compatibility of biological interfaces and enhance the efficiency of cell signal exchange in the artificial microenvironment.

In addition to introducing cell recognition sequences, bioactive molecules can also be immobilized within hydrogels by covalent or non-covalent interactions to achieve localized sustained release and precise controlled release [92]. For example, the stable combination of vascular endothelial growth factor (VEGF) with hydrogel can continuously promote angiogenesis in wound repair, support capillary sprouting and improve tissue perfusion [93]. In bone regeneration, the active fragments of bone morphogenetic protein-2 (BMP-2) are directly conjugated to polypeptides or covalently connected hydrogel, which can significantly promote osteoblast differentiation and matrix mineralization, and accelerate bone tissue repair [92]. Similarly, loading AMPs in hydrogel can inhibit bacterial colonization at the implantation site, mitigate the risk of infection, and improve the long-term stability of the material [11]. Such a functional design makes peptide hydrogels not only a structural scaffold, but also a local sustained-release drug delivery system for local treatment.

In summary, the biofunctional design makes the peptide hydrogels not only have stable structural strength, but also have precisely regulated biological activity. As a dynamic microenvironment carrier, such materials can actively regulate cell behavior and molecular interaction, and are widely used in tissue repair, organ regeneration and clinical implantation. With both mechanical properties and tailored biological functions, it is a new generation of therapeutic scaffolds with great potential in the field of regenerative medicine. For clarity and better understanding, the major biofunctionalization approaches and their representative biomedical applications are summarized in Table 2.

### 3.3. Tunable Physical Properties

The physicochemical characteristics of peptide hydrogels directly determine their performance in tissue engineering and regenerative medicine. Therefore, in the design stage, it is necessary to accurately regulate the network structure, mechanical stiffness, viscoelasticity, pore morphology and other parameters [28,101]. Matching the structure and rheological performance according to the microenvironmental needs of the target tissue can achieve better functional integration, support cell behavior, and ultimately improve the therapeutic efficacy.

Mechanical properties are the core functional determinants of peptide hydrogels. By adjusting the proportion of hydrophobic and hydrophilic amino acids in the peptide sequence, the accumulation density of self-assembled nanofibers can be finely regulated and the stability of the intermolecular hydrogen bond network can be enhanced [27,102]. This nanoscale modification can directly macroscopically regulate the elasticity of the material, so that the hydrogel modulus matches the mechanical needs of the target tissue. Low-modulus compliant matrices are more suitable for soft tissue repair such as fat and nerves, and smaller mechanical resistance is conducive to tissue integration, while high-modulus rigid networks are suitable for cartilage and bone regeneration, which can withstand continuous mechanical loads and ensure long-term functional stability [102].

Viscoelasticity directly determines the behavior of cells in hydrogels and regulates key processes such as cell migration, proliferation and mechanical signal conduction [103,104]. The cell morphology is more stable in the highly viscoelastic matrix, and the random migration is limited, which is conducive to maintaining the tissue morphology in the scaffold [28]. Low viscoelastic modulus materials can improve cell activity and promote the ECM remodeling, which has significant advantages in the regeneration process of wound healing, blood vessel sprouting and tissue maturity [105]. Therefore, rational tuning of viscoelastic parameters can balance structural stability and biological adaptability, and optimize the scaffold performance according to treatment needs [28].

Pore structure is the critical parameter affecting the function of hydrogels, which is directly related to nutrient transport, oxygen penetration, metabolic waste discharge and drug release rate [106,107]. Precise control over hydrogel porosity can be realized by introducing sterically bulky groups into peptide sequences, which regulates the assembly behavior of nanofibers. Alternatively, tailored chemical and physical crosslinking during gel formation allows fine adjustment of pore size, structure, and spatial distribution within the network [108]. In scenarios that require a large number of cells to invade, such as bone repair, large-sized interconnected pores can promote cell migration and angiogenesis, while the sustained-release drug delivery system is more suitable for small and uniform pores, which can extend the drug action time by limiting diffusion and maintaining local stable concentration [109,110]. Rational tuning of the pore structure can make the hydrogel accurately match the needs of different biomedical applications.

Injectability is the key performance of the clinical application of peptide hydrogels, which is especially suitable for the treatment of deep and irregular lesions [111]. The ideal injectable system has low viscosity and good fluidity at room temperature, and can be injected smoothly through fine-gauge needles; when exposed to physiological conditions such as body temperature, ionic strength or pH, it can quickly gel to form a stable in situ scaffold [24]. This balance requires precise sequence design to make the material have sensitive stimuli-responsive self-assembly ability, maintain liquid state during injection, and quickly form a crosslinked network after reaching the target [16]. This characteristic can effectively reduce surgical trauma, improve the accuracy of treatment positioning, and significantly improve the patient prognosis [111].

### 3.4. Controlling Degradability and Immunogenicity

Degradability is the critical parameter of the adaptability of peptide hydrogels in vivo. If materials remain at implantation sites for a long time, it is easy to cause chronic inflammation, fibrosis, and even hinder tissue regeneration. To achieve the ideal therapeutic efficacy, it is necessary to accurately regulate the degradation rate to match it with the regeneration cycle of the target tissue [112]. For short-term treatment such as acute skin trauma, hydrogels should be gradually degraded, usually completing metabolic clearance within a few weeks [24]. On the other hand, long-term applications such as bone repair and cartilage reconstruction are the opposite. The scaffold is required to maintain structural integrity for an extended period of several months, and subsequently undergo slow, controlled degradation. This long-term support can ensure that sufficient mechanical support is provided before the new tissue is fully formed and replaces the material [113].

Most hydrogels mainly rely on specific enzymatic reactions to achieve degradation. The introduction of enzyme-cleavable motifs that can be cleaved by matrix metalloproteinase (MMPs), lysozyme, elastase, etc., into the main chain of polypeptides can gradually decompose the scaffold in the physiological environment [114,115]. This well-controlled degradation process yields non-toxic polypeptide fragments and amino acids that are readily metabolized and subsequently eliminated from the body. This method not only avoids the accumulation of toxic byproducts but also improves the long-term biosafety and compatibility of materials.

Immunogenicity control is crucial to the clinical translation of peptide hydrogels. An exacerbated immune response will cause the scaffold to be quickly removed by the body, or cause acute and chronic inflammation, which will ultimately affect the therapeutic efficacy [116]. Reducing immunogenicity needs to start from molecular design, so that the peptide sequence is closer to the human endogenous proteins, reduce xenogeneic recognition, and improve immune tolerance [117]. At the same time, minimize the surface exposure of highly immunoreactive residues such as aromatic side chains and strongly charged amino acids, reduce non-specific immune activation, and improve the long-term biocompatibility of materials [94].

In addition to peptide sequence optimization, targeted immunomodulation functionalization can further avoid host adverse reactions. The introduction of corticosteroid conjugates, anti-inflammatory short peptides and other biologically active molecules in hydrogels can selectively inhibit the pro-inflammatory signaling pathway, reduce the release of cytokines and reduce the activation of immune cells [8]. Through such precise molecular modification, peptide hydrogels can improve biocompatibility, maintain structural and functional stability in vivo, and ensure the therapeutic effect without causing harmful immune reactions [100]. This strategy can establish continuous immune tolerance in vivo and provide reliable support for safe and stable clinical translation.

## 4. Structural Characteristics and Properties of Self-Assembling Peptides

On the basis of the self-assembly mechanisms and modular design principles described above, the molecular sequence and assembly behavior collectively endow peptide hydrogels with unique multi-scale structural and mechanical features. The nanostructure and micromorphology of self-assembled peptides lay the foundation for network construction, while the porous characteristics of three-dimensional networks govern mass transport and cell infiltration behavior. Meanwhile, mechanical performances including elasticity, strength and viscoelasticity further determine the service stability and tissue adaptability of hydrogels in physiological microenvironments. To clarify the inherent structure–property correlation, this section systematically summarizes the typical nanostructural forms, porous architecture features, and tunable mechanical characteristics of programmable peptide hydrogels.

### 4.1. Nanostructures and Micromorphology

The functional properties of peptide hydrogels are directly determined by their internal nanostructure and micromorphology. During the self-assembly process, the polypeptide chain stabilizes the formation of one-dimensional or two-dimensional nanostructure units through non-covalent interactions such as hydrogen bonding, hydrophobic interactions, and aromatic side chain π–π stacking [18]. These primary units can be further assembled by spatial entanglement, parallel alignment, or helical coiling, and finally construct a three-dimensional crosslinked hydrogel network [118]. This hierarchical architecture not only determines the overall mechanical properties of the material, but also significantly regulates the behavior of cells in the matrix, including adhesion, directional migration and response to external physicochemical signals [119].

One-dimensional nanofibers are the most common among the various structures formed by self-assembled polypeptides. The diameter of these fibers is mostly a few nanometers to tens of nanometers, and the length can reach tens of microns [80]. Continuous long fibers can be seen under electron microscopy and atomic force microscopy, with a prominent aspect ratio, and the structure closely mimics the natural ECM [120], which can improve cell adhesion and guide cells to migrate along the fiber axis. In contrast, the two-dimensional nanosheets structure is mostly composed of stacked β sheets, which have a large specific surface area and are rich in functional groups [30]. They can load bioactive sites with high density and realize local strong biochemical signaling, and have an outstanding effect on inducing neuron differentiation and regulating stem cell directional differentiation [121].

Some special self-assembly paths can form a hollow nanotube structure, and its internal cavity can load small molecule drugs, signal peptides or metal ions [122]. The enclosed cavity can not only physically protect the load and improve molecular stability, but also realize precise site-specific release in the physiological environment of the body. Therefore, nanotube hydrogels are suitable as a general carrier platform for targeted drug administration, molecular imaging and stimulus-responsive treatment systems [123].

Different nanostructures give peptide hydrogels unique macroscopic and functional properties. Fiber networks can closely mimic the three-dimensional structure of the ECM, which is conducive to cell adhesion, spreading and directional migration. Due to the density of surface functional groups, the plane sheet assembly is more suitable for the directional differentiation process of cells that depends on continuous local biochemical signals [124]. At the same time, the nanotube structure is extremely applicable to the encapsulation and controlled release of small molecule drugs and protein drugs [117]. At the design stage, the nanomorphology and hierarchical assembly methods are precisely regulated, and the corresponding mechanical, biochemical and biological properties of hydrogel can be tailored to meet the diversified needs of tissue engineering and regenerative medicine. Table 3 summarizes the typical fabrication methods and their porous characteristics and physical properties, which is convenient for comparison and consultation.

### 4.2. Porous Characteristics of Three-Dimensional Networks

After the formation of the three-dimensional hydrogel network, the internal pore structure directly determines the performance and biocompatibility of the material. Key parameters such as pore size, distribution uniformity and interconnectivity not only affect the diffusion of water, nutrients and gas, but also regulate the arrangement, migration and metabolic activity of cells in the matrix [125]. In tissue engineering, the ideal pores are multi-level and multi-scale structures: micropores are responsible for efficient nutrient exchange and waste removal, while mesopores and large pores of appropriate sizes provide space for cell infiltration, proliferation and tissue remodeling [129]. Only by rationally tuning the pores of different scales can we maintain the biological activity of the scaffold while maintaining the mechanical stiffness and structural stability.

In the design stage, the pore structure of hydrogels can be precisely regulated by introducing sterically bulky amino acid side chains into the main chain of polypeptides. Such a residue with steric hindrance alters the molecular stacking method and guides the fiber structure to complete self-assembly according to the preset pore size distribution [19]. Using a hierarchical self-assembly strategy, it can regulate the structural characteristics of nanometer and micron scales at the same time, and prepare pore structures with diverse functions and specific forms [130]. In addition, the crosslinking method has a significant impact: low-density crosslinking forms a high-permeability network, which is conducive to deep infiltration and migration of cells, but the mechanical stiffness is low; high-density crosslinking has excellent mechanical properties and load-bearing, but it will limit the penetration of macromolecules and drugs [131].

At present, a variety of pore engineering technologies have been applied to hydrogel preparation to further enhance their function. For example, the freeze-drying method forms uniform large pores through the ice crystal template in the dehydration process; the gas foaming method uses instantaneous bubbles in the gel process to build a highly connected mesoporous and large pore network [132,133]. Both methods can significantly improve the porosity of large pores without destroying the original nanostructure, so that the hydrogel has efficient mass transport ability and stable mechanical properties at the same time [134]. Such an optimized pore structure has obvious advantages in high-demand regeneration scenarios such as cartilage repair. The scaffold can balance high permeability and sufficient bearing capacity [23,135].

### 4.3. Mechanical Properties

The mechanical properties of peptide hydrogels (elastic modulus, yield strength, viscoelasticity, etc.) directly determine their structural support ability and biological regulation effect in vivo. These mechanical properties affect the direction of cell adhesion, migration and differentiation, and also determine the degree of fusion between the material and the host tissue and the long-term stability under physiological stress [103]. Low-modulus, high-water-content hydrogels can mimic the mechanical properties of the ECM of soft tissues such as brain and heart muscle, which is conducive to cell adhesion, proliferation and morphology maintenance [136]. The high modulus system is more suitable for hard tissue engineering such as bone and cartilage repair. Its continuous bearing capacity is the key to long-term functional stability [23].

The mechanical behavior of peptide hydrogels is mainly determined by the crosslinking of nanofibers and the supramolecular network, and the two are finely regulated by the amino acid composition and the hydrophobic balance in the peptide sequence [30]. When phenylalanine, tyrosine and other aromatic residues are enriched in the sequence, the molecular core can be tightly stacked through π-π stacking, which significantly improves the elastic modulus and yield strength [122]. On the contrary, the introduction of conformational flexible residues such as glycine and proline will reduce packing density, increase the space for molecular rearrangement, and improve the viscoelasticity of the material while reducing rigidity [28,86].

**Figure 3:**
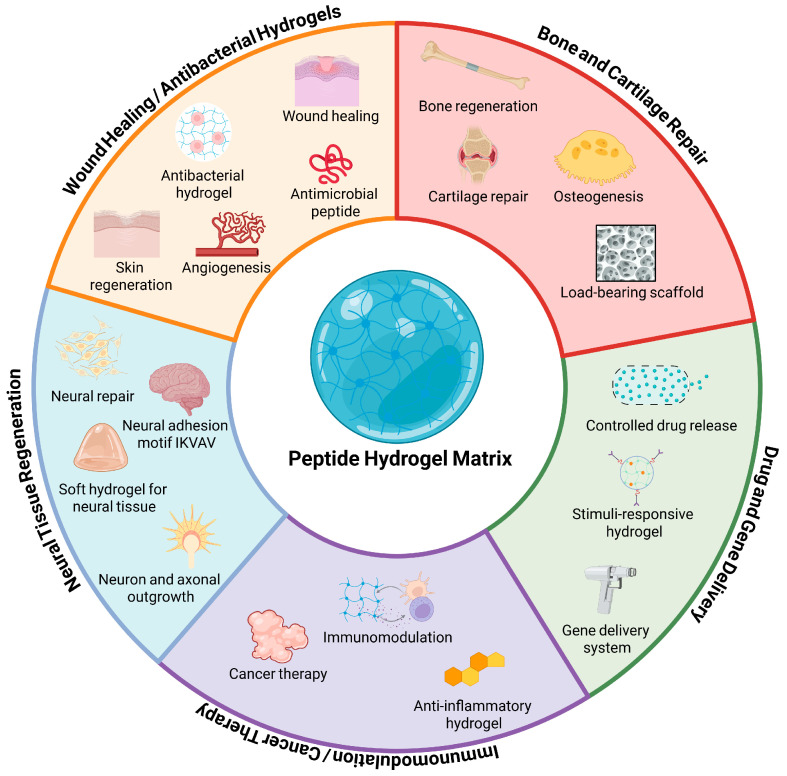
Representative biomedical applications of self-assembling peptide hydrogels. Self-assembled peptide hydrogels are a kind of widely used biomedical carrier material. Peptide hydrogels exhibit diverse biofunctions in various biomedical applications. They promote wound healing by enhancing skin regeneration, stimulating angiogenesis, and exerting antibacterial activity. They facilitate bone and cartilage repair through osteogenic differentiation and load-bearing scaffold construction. In addition, they support nerve tissue regeneration by promoting neuronal growth and adhesion, enable controlled drug and gene delivery via stimuli-responsive systems, and exert anti-inflammatory effects that contribute to immune regulation and tumor therapy. Created in BioRender. Zhao, Y. (2026), https://BioRender.com/mbyuune (accessed on 22 April 2026).

The hierarchical relationship between the primary sequence, microstructure and macroscopic mechanical properties of peptides fully reflects the core role of rational peptide design. By reasonably regulating the residue composition and self-assembly conditions, the hydrogel mechanical properties can be accurately matched with the biomechanical microenvironment of the target tissue. This sequence-oriented mechanical regulation can customize the material performance on demand to meet the diversified application needs of tissue engineering and regenerative medicine (Figure 3).

## 5. Biofunctional Applications

Based on the well-defined nanostructures, porous architectures, and tunable mechanical properties systematically elaborated above, programmable peptide hydrogels integrate biomimetic three-dimensional support, responsive release capability, and diverse bioactive functions, making them versatile platforms for multifunctional biomedical applications. Benefiting from their sequence-customizable features and microenvironment-adaptive performance, these supramolecular materials have been extensively exploited in multiple cutting-edge clinical-oriented fields. This section comprehensively summarizes their representative advances in tissue engineering and regenerative medicine, targeted drug and gene delivery, cell-based therapy and immunomodulation, as well as antibacterial and antiviral biomedical scenarios.

### 5.1. Tissue Engineering and Regenerative Medicine

In recent years, functional peptide hydrogels have become a material platform with strong adaptability and high clinical value for three-dimensional (3D) cell culture, tissue engineering and regenerative medicine. Such a biological material is not simply a passive structural support, but can actively construct a molecular microenvironment and accurately mimic the physical, chemical and biological characteristics of natural ECMs [137]. Through the rational design of amino acid sequences and the precise regulation of the self-assembly process, a microenvironment that is highly close to the physiological state can be built, support cell proliferation, guide cell differentiation, and maintain long-term functional stability [138]. Compared with traditional scaffolds, peptide hydrogels can efficiently construct a stable three-dimensional cell structure in vitro, and can also directly form the structure required for regeneration in situ of biological tissue, simplify the preparation process and reduce invasive implantation operations [111]. These advantages simplify the process of regenerative medicine, improve the operation accuracy and therapeutic efficacy, and further establish the status of peptide hydrogels as a high-performance carrier material in advanced biomedicine.

In the three-dimensional culture system, maintaining cell activity and function requires not only a suitable spatial structure, but also continuous signal molecules to regulate cell adhesion, migration, differentiation orientation and functional maintenance. The core advantage of peptide hydrogels lies in their dual characteristics: they can not only provide mechanically stable and structurally regulated scaffolds for cell adhesion and colonization, but they also provide biochemical signals simultaneously [139]. RGD, IKVAV, YIGSR and other classic adhesion sequences, as well as polypeptide fragments that can be combined with growth factors, can be directly connected to the peptide backbone to accurately regulate the fate of cells without destroying the hydrogel structure [140,141]. On this basis, SDF-1, IL-4 and other multifunctional sequences are introduced, which can simultaneously regulate cell proliferation, migration and differentiation at the same time. Studies show that hydrogel functionalized by SDF-1/IL-4 can significantly promote fibroblast proliferation and endothelial cell activation in muscle tissue remodeling, and there is no obvious cytotoxicity, showing the application potential of dual-function regeneration scaffolds [142].

**Figure 4:**
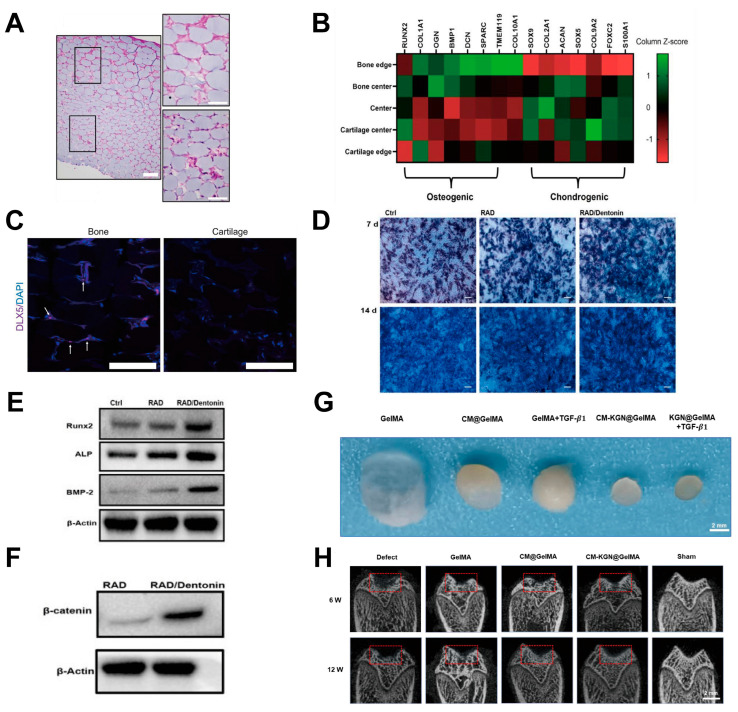
The representative results of many studies reflect the regeneration effect of peptide hydrogels in bone cartilage repair. (**A**) Osteological observation: Tissue morphology and integration of defective parts. (**B**) Heat map illustrating the expression profiles of osteogenic and chondrogenic markers in various tissue regions. (**C**) Immunofluorescence: Cell localization and differentiation in bone and cartilage areas. (**D**) Alizarin red staining: Mineralized sedimentation after 7 days and 14 days of culture. (**E**,**F**) Western blot detection: Expression of bone-related proteins (Runx2, ALP, BMP-2) and signaling proteins (β-catenin). (**G**) Macro-morphology after different hydrogels (GelMA, CM@GelMA, CM-KGN@GelMA, with/without TGF-β1). (**H**) Microscopic CT reconstruction: Bone regeneration after 6 weeks and 12 weeks of implantation. Panels (**A**–**C**) reproduced from [3]. Panels (**D**–**F**) reproduced from [144]. Panels (**G**,**H**) reproduced from [143].

At the same time, self-assembled polypeptide systems such as RAD equipped with dentin can form a regular β sheet supramolecular structure, which can significantly promote vascularized bone repair and is suitable for cranial defect regeneration [143]. Histological observation shows that these multifunctional peptide hydrogels enable the seamless integration of bone and cartilage tissues, forming a continuous tissue interface with blurred boundaries (Figure 4A). Transcriptomic analysis shows that there are spatial differences in the expression of osteogenic and cartilage-related genes in the bone–cartilage junction, indicating that the direction of cell differentiation is regulated by the microenvironment (Figure 4B). Immunofluorescence results show that DLX5-positive cells are mainly concentrated in the bone region, suggesting that osteogenic differentiation and matrix remodeling are significantly enhanced (Figure 4C) [3]. In another model, RAD/dentin hydrogel can quickly form a mineralized matrix, and the alkaline phosphatase activity and calcium deposition on the 7th and 14th days were significantly higher than those of the control group (Figure 4D). Protein detection further confirmed that Runx2, ALP and BMP-2 were significantly upregulated, and the β-catenin pathway was activated, indicating that the osteogenic transcriptional cascade was activated, and the efficiency of stem cell differentiation was improved (Figure 4E,F) [144].

In recent years, advances in the functional modification of stimuli-responsive systems have allowed the performance of hydrogels to be regulated with greater precision, both dynamically and in situ. Hydrogels with matrix metalloproteinase cleavable sequences can realize the localized release of drugs in the microenvironment of bone tissue inflammation, effectively deal with the problem of microenvironmental heterogeneity, and achieve targeted repair. Nano-hybrid peptide hydrogels prepared via hierarchical self-assembly exhibit superior adaptability, enabling real-time regulation of fibrous cartilage architecture and simultaneous regeneration of tissue structure and function [145]. Macroscopic observation shows that the scaffold structures of CM@GelMA and CM-KGN@GelMA are stable, which can support cell aggregation and promote cartilage remodeling (Figure 4G). The results of continuous microscopic CT consistently show that after 6 and 12 weeks of implantation, the osteochondral defect is gradually filled, the trabecular microarchitecture is repaired, and a large volume of robust subchondral bone is generated and seamlessly integrated with the surrounding normal tissue (Figure 4H) [143].

The core feature of peptide hydrogels is their molecular programmability coupled with highly controlled physical tunability. Sequence modifications permit nanoscale adjustments in parameters such as stiffness, pore size distribution, and surface chemistry, while variations in crosslinking density or peptide concentration allow the elastic modulus to be matched precisely to the biomechanical profile of the intended tissue [91]. Owing to their high water content, these systems are flexible and facilitate rapid diffusion of nutrients, metabolites, and oxygen, properties that are essential for sustaining complex 3D cellular constructs [138]. In myocardial tissue engineering, peptide hydrogels have both mechanical support and angiogenesis, which can promote myocardial function recovery and integration with host tissue.

The biofunctionalization of peptide hydrogels is the core means to improve their biological activity. The common method is to introduce adhesion peptide sequences to mediate receptor-dependent cell interactions and activate downstream signaling pathways [13,137]. Among them, a large number of RGD tripeptides in natural ECMs are crucial to promote cell adhesion and maintain cytoskeleton stability, while IKVAV and YIGSR sequences derived from layered adhesion proteins can effectively support neuronal adhesion, axon extension and cell differentiation [121,146]. Research has confirmed that connecting to the IKVAV substrate in the cryogel embedded with polycaprolactone (PCL) can fabricate a biomimetic scaffold, effectively guide the regeneration of peripheral nerves, and arrange neurons in an orderly manner to form a regular three-dimensional network [86,121].

In recent years, the biofunctionalization of peptide hydrogels has broken through the traditional sequence modification, and strategies such as co-assembly, peptide–polymer compounding and advanced biomanufacturing integration have been developed. Such a composite method takes into account the structural strength of synthetic polymers and the bio-specificity of polypeptides, and can accurately regulate cell–matrix interactions, signal transmission and tissue remodeling. In additive manufacturing, polypeptide functionalized hydrogels are suitable as a high-performance biological ink, containing living cells and growth factors, and building a tailored regeneration structure, which is especially suitable for cartilage repair [147]. In cartilage engineering, co-assembled bone peptide systems loaded with GDF5 (such as P11-4, P11-8) can significantly accelerate the aggregation and tendon differentiation of progenitor cells, shorten the repair cycle and promote tissue maturation [23]. In terms of nerve regeneration, the precisely designed peptide hydrogels have improved biocompatibility, which can guide axon growth and improve repair efficiency, and have a promising translational potential in complex tissue reconstruction [148].

Accumulating evidence from both animal models and early-stage clinical studies highlights the broad regenerative capacity of peptide hydrogels across diverse tissue repair contexts. In osteochondral defect reconstruction, a particularly promising approach has involved the fabrication of bilayer or gradient hydrogel systems in which an N-cadherin-derived functionalized interface promotes chondrogenic signaling and cell aggregation, while a complementary osteo-inductive layer incorporates bioactive peptides such as Gly-His-Lys (GHK) or small-molecule chondrogenic agents to stimulate bone remodeling [94]. In rabbit femoral condyle defect models, such programmable constructs—often combined with stimuli-responsive features to modulate macrophage polarization or release growth factors on demand—have yielded marked improvements in cartilage surface regularity, enhanced glycosaminoglycan (GAG) deposition, accelerated subchondral bone matrix infiltration, and seamless integration of the osteochondral junction within a twelve-week period [23,149]. Comparable dual-gradient or composite hydrogels, including CM-KGN@GelMA systems, have likewise reproduced native tissue architecture and biomechanical performance in vivo.

**Figure 5:**
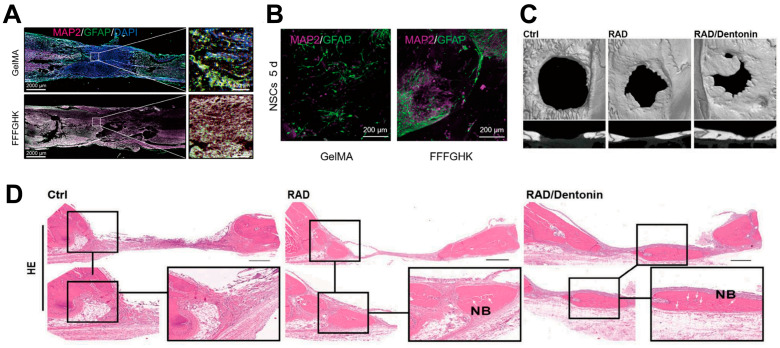
Composite illustration of biomaterial hydrogel performance in neural, bone, and muscle tissue regeneration. (**A**) Representative immunofluorescence images of neural tissue stained with MAP2 (purple), GFAP (green), and DAPI (blue) on GelMA and FFFGHK hydrogel scaffolds. (**B**) Confocal observation showing NSC adhesion and differentiation after 5 days of culture on both hydrogels. (**C**) Micro-CT and morphology of regenerated bone defect under different conditions (Ctrl, RAD, RAD/Dentonin). (**D**) H&E staining of repaired tissue demonstrating new bone (NB) formation in different treatment groups. Panels (**A**,**B**) reproduced from [150]. Panels (**C**,**D**) reproduced from [143].

Within the central nervous system repair domain, recent advances have shifted toward bioactive self-assembling peptide hydrogels platforms incorporating motifs such as GHK or N-cadherin mimetics, which emulate ECM-like microenvironments and provide sustained neurotrophic support. Engineered to respond to external cues—ranging from magnetic stimulation to localized enzymatic activity—these matrices can amplify regenerative signaling, guide axonal projection, and enhance synaptic connectivity. In vivo evaluation demonstrated that the FFFGHK peptide hydrogels distinctly enhanced neural tissue bridging and axonal continuity across the lesion zone compared with the unmodified GelMA scaffold, accompanied by denser MAP2^+^ neuronal fibers and reduced reactive gliosis, indicating robust neuronal maturation and reinnervation within six days of implantation (Figure 5A). The results of neural stem cell immunofluorescence also confirm that the FFFGHK matrix is more conducive to the growth of neurite growth and the regular arrangement of glial cells. The cell–matrix interaction is better, and it has a good microenvironment to promote nerve development (Figure 5B) [150]. In in vitro experiments, these hydrogels can support the induction of pluripotent stem cells (iPSCs) to form cortical organs, significantly promote axon extension and synapse generation, and improve the structural complexity and functional maturity of neural networks [151]. In the spinal cord injury model, functionalized peptide hydrogels can effectively improve the recovery of motor function and promote the reconstruction of neural circuits, and they have clinical translation potential in a variety of CNS diseases [152,153].

In bone tissue engineering, PdBT-derived crosslinked scaffolds functionalized by BMP-2 mimetic peptides can significantly improve bone induction ability, which is manifested as increased alkaline phosphatase (ALP) activity of osteoblasts and rapid deposition of mineralized matrix. In vivo experiments also confirmed that the scaffolds can accelerate the bridging of bone defects, form a mechanically stable bone–implant interface, and effectively promote bone integration [143,154,155]. On this basis, the new programmable peptide hydrogel realizes multi-signal coordination and immune microenvironment regulation. For example, 3D-printed microsphere–hydrogel hybrid constructs engineered for temporally controlled release of NGF-mimetic and BMP-2 peptides can simultaneously activate CGRP-dependent neuronal pathways and osteogenic differentiation in mesenchymal stromal cells, achieving vascularized bone regeneration in critical cranial defect models [156]. μCT and histological analyses of critical-size bone defects revealed that the RAD/Dentonin hydrogel promoted substantial formation of neobone tissue with uniform mineralized continuity compared with RAD alone or untreated controls. The repaired sites exhibited well-integrated trabecular networks and minimal fibrotic gaps, signifying effective osteoconduction and defect bridging (Figure 5C). H&E staining further illustrated complete filling of the defect cavity and homogeneous bone matrix deposition, confirming spatially directed osteogenic recruitment and rapid bone remodeling (Figure 5D) [143]. Likewise, self-assembling RAD/Dentonin peptide hydrogels enhance BMSC osteogenesis by triggering Wnt/β-catenin signaling and boosting paracrine endothelial recruitment [143], advancing strategies for maxillofacial vascularized reconstruction. Notably, peptide conjugation via click chemistry preserves bioactivity more efficiently than glutaraldehyde crosslinking, thereby fostering capillary network formation and PI3K–AKT pathway activation in ischemic niches, while ultrasound-responsive nanofiber hydrogels permit on-demand M2 macrophage polarization and sequential secretion of BMP-2/IGF-I to re-establish a pro-osteogenic immune microenvironment [157].

In parallel, peptide-based bioengineered scaffolds have been used in skeletal muscle regeneration, and the research has gradually expanded from cell-laden systems to cell-free strategies and matrix biomechanical regulation. The introduction of laminin-derived Q polypeptide (RKRLQVQLSIRTC) into gelatin hydrogel can create a permissive microenvironment for skeletal muscle cells and promote muscle fiber regeneration and angiogenesis in the muscle injury model [158,159]. After 8 weeks of implantation, α actinin and myosin heavy chain (MHC) were significantly upregulated, and the morphology and function were significantly recovered [160]. Porous DGL/PEG peptide hydrogels can be injected as an acellular scaffold. After stiffness optimization, they can promote the adhesion, proliferation and directional arrangement of muscle cells, and the orderly anchoring and repair of muscle fibers can be realized within 21 days in the rat muscle defect model [161]. In tendon–bone interface repair, Mg/BMP-12 polypeptide thermosensitive gel can achieve sequential release: free magnesium ions are anti-inflammatory in the early stage, and chelated magnesium ions regulate immunity in the later stage, synergistically improving the interface regeneration effect [162]. In addition, hyaluronic acid hydrogel with hFAPs, bsp-RGD(15) peptides and heparin can inhibit fibrosis and fat infiltration, while upregulating UCP1 and IL-10, and significantly improving muscle mass after 6 weeks [163].

In wound repair and scar regulation, the hyaluronic acid hydrogel dressing loaded with AMP DP7 can achieve high-efficiency scar-free healing in the deep burn model [164]. On this basis, programmable peptide/hyaluronic acid hydrogel prepared by two-step crosslinking, which has both angiogenesis and antibacterial ability, can provide structural support and bioactive regulation in burn repair at the same time [165]. Smart sequential control strategies have also emerged, exemplified by dopamine- and sulfonated-HA-modified lubricating hydrogels loaded with copper single-atom nanozymes and KGN, which reduce oxidative stress and provide sustained chondrogenic cues—a mechanism applicable to chronic wound inflammation-to-regeneration transitions [166]. Similarly, HA methacrylate composite hydrogels conjugated with trivalent β-peptides (HA-β3Tc) exhibit >90% TGF-β1 sequestration combined with curcumin-mediated NF-κB suppression, orchestrating early anti-inflammatory signals followed by regenerative factor enrichment to expedite closure and attenuate fibrosis [167]. In another modality, cryo-structured HA hydrogels embedding DP7 and PMSCs (DA7CG) coordinate infection suppression in the inflammatory phase, stimulate skin, vascular, and follicular regeneration during proliferation, and modulate ECM remodeling in the maturation phase, resulting in full-cycle, scar-less tissue restoration [164,168].

Within the rapidly evolving domain of 3D tumor modeling, programmable peptide hydrogel systems are increasingly recognized for their capacity to couple mechanically tunable architectures with biochemically precise signaling modules, thereby enabling both high-resolution interrogation of tumor microenvironmental dynamics and the execution of high-throughput therapeutic screening. For instance, peptide amphiphile PA-E3Y hydrogels can be engineered with crosslinking density gradients to replicate stromal stiffness heterogeneity typical of desmoplastic malignancies such as pancreatic ductal adenocarcinoma, subsequently inducing extracellular matrix deposition, epithelial–mesenchymal transition (EMT), CD133^+^/CXCR4^+^ cancer stem cell enrichment, and enhanced chemoresistance—features emblematic of aggressive in vivo phenotypes [169]. In colorectal carcinoma models, PEG-based programmable hydrogel matrices have elucidated stiffness-dependent growth patterns, wherein softer matrices (~300 Pa) promote multicellular spheroid formation and stiffer matrices (~2 kPa) suppress proliferation, while integrin-specific adhesive peptides differentially modulate EGFR-mediated downstream cascades, highlighting synergism between biomechanical cues and biochemical signaling [170].

Modular incorporation of bioactive peptide sequences enables platforms with finely tuned biological outputs. Laminin-mimetic IKVAV peptides within hyaluronic acid–tyramine (HA-Try) hydrogels of adjustable stiffness (0.69–2.24 kPa) have been shown to sustain brain organoid development, with morphological and multi-omics profiles closely resembling those obtained in Matrigel, thereby offering a chemically defined, xeno-free alternative for neural tumor and organoid modeling [171]. Dual-peptide conjugation targeting α6β1/α2β1 or αVβ5 integrin only require extremely low concentrations to achieve high-efficiency cell adhesion and functional regulation, which is better than the traditional high-dose system and has better repeatability [172]. Similarly, sulfated self-assembling peptides that mimic heparan sulfate S-domains can specifically bind and sequester VEGF in the tumor microenvironment to inhibit angiogenesis and invasion, which is a typical application of the “negative regulation” of microenvironment signals [173].

The stimulus-responsive design can mimic the pathological microenvironment more realistically and dynamically. The ROS-responsive hydrogels modified by ferrocene can release hydrophilic doxorubicin and hydrophobic paclitaxel respectively, which can effectively inhibit the growth of melanoma in an oxidative stress environment, and the minimal off-target toxicity [174]. It contains KK sequence and other pH-responsive short peptides, which can stabilize the load of paclitaxel in a neutral environment and achieve sustained release in the weak acidic microenvironment of the tumor, enhance antitumor efficacy, reduce the systemic toxicity, and accurately mimic the acidic microenvironment in drug screening [79]. In another complementary strategy, dual enzyme-responsive peptide precursors can self-assemble into nanofibers in specific tumor cells, helping drugs escape from lysosomes and achieve targeted chemotherapy delivery; combined drugs can also enhance the effect of immunotherapy [77].

From the perspective of clinical translation, by combining the patient-derived tissue with the peptide hydrogels to mimic the ECM, and with high-throughput 3D bioprinting, a model of children’s neuroblastoma and sarcoma-like organs has been constructed, which can fully preserve the genome and phenotype characteristics of the primary tumor and be used for individualized drug sensitivity screening [175]. Self-assembled SCIBIOIII peptides can form a dense nanofiber network, which can stably support the three-dimensional culture of colorectal adenocarcinoma and provide a platform for personalized anti-tumor drug screening [176]. In addition, the introduction of peptide stereo-complex crosslinking agent in PEG hydrogel can significantly improve the resistance to proteolytic degradation. The structural integrity of protease K still reaches 80% after 1 h of processing, and the mechanical properties can be restored by 50–70% after cyclic load, which improves long-term stability and in vivo applicability [177]. In summary, the combination of programmable mechanical properties, reasonable bioactive peptide design, stimuli-responsive function and patient-derived materials has greatly improved the physiological simulation and translational value of 3D tumor models.

In summary, peptide hydrogels have developed into a fully functional biomimetic biomaterial, which can accurately regulate cell behavior in a complex three-dimensional microenvironment. They have both a stable mechanical structure and regular geometric form. With spatiotemporally controlled biochemical signals, it can be well integrated with the host tissue and initiate the collaborative regeneration process in multi-organ tissues. The material is highly programmable, can adjust the stiffness and degradation rate, and modularly carry the matrix sequence, which can closely mimic the dynamics of the natural extracellular matrix and adapt to physiological environment changes in real time. In the future, the new generation of peptide hydrogels will focus on the precise regulation of multi-signal coordination and biological functions in time and space to achieve the optimal matching of structural stability and biological functions.

### 5.2. Drug and Gene Delivery

The application of functional peptide hydrogels in drug and gene delivery has garnered considerable attention. Their molecular structures are rationally designable, the three-dimensional network is mechanically stable and adjustable, and the biocompatibility is excellent. Such a multifunctional carrier can simultaneously introduce targeted peptide sequences to achieve high-efficiency drug loading and have the characteristics of stimuli-responsive degradation. Such an integrated design enables the optimization of competing therapeutic requirements within a single construct, thereby significantly improving delivery precision, enhancing therapeutic efficacy, and expanding the applicability of advanced biomedical interventions [7].

**Figure 6:**
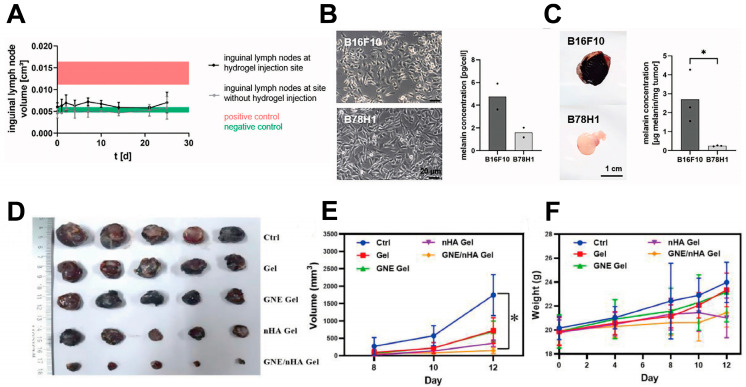
Targeted and stimuli-responsive hydrogel systems for localized therapeutic delivery. (**A**) Changes in the volume of groin lymph nodes in rats after local injection of hydrogel indicate good biocompatibility of hydrogel. (**B**) Comparison of the morphology of melanoma cells (B16F10 and B78H1) and their cellular melanin content. (**C**) The differences in melanin between different tumor tissues and their quantitative analysis reflect the differences in the characteristics of model tumors, *: p < 0.05. (**D**) Digital photographs of excised tumors after various treatments. (**E**) Quantitative analysis of tumor volume changes at different treatment time points, *: p < 0.05. (**F**) Changes in body weight of mice receiving different treatments. Panels (**A**–**C**) reproduced from [178]. Panels (**D**–**F**) reproduced from [179].

Programmable peptide hydrogels are a new generation of stimuli-responsive delivery systems, which can realize the accurate release of therapeutic drugs in time and space through the molecular design and adaptive network structure. The system can simultaneously load hydrophilic drugs such as interleukin-4 (IL-4) and hydrophobic drugs such as paclitaxel (PTX) in the three-dimensional matrix, and achieve accurate co-delivery according to local biochemical signals. This structure effectively inhibits the burst release of drugs, ensures the sustained release of drugs at the tumor site, significantly improves the accuracy of treatment and reduces systemic toxicity. The programmable system has excellent biocompatibility and local controlled release performance. After hydrogel administration, the volume of inguinal lymph nodes is stable, the inflammatory reaction is mild, and it has good safety and immune tolerance (Figure 6A). In vitro experiments show that it can achieve high-efficiency drug uptake and metabolic regulation in melanoma cells (B16F10, B78H1). The delivery of IL-4 and PTX can induce cellular remodeling and inhibit pigmentation, reflecting a good targeting effect (Figure 6B). Tumor tissue sections show characteristic pigment distribution due to the difference in local controllable accumulation and degradation kinetics of drugs, reflecting the programmed response of hydrogel in the lesion microenvironment and confirming the spatial and temporal accuracy of drug release (Figure 6C) [178].

Functional peptide hydrogels can accurately respond to the biochemical characteristics of the pathological microenvironment, such as high expression of protease, abnormal pH and excessive production of reactive oxygen species (ROS) [180]. The introduction of matrix metalloproteinase (MMP) substrate sequences (such as PLGLAG sensitive to MMP-2/9) can make hydrogels degrade at the tumor or inflammatory site at a fixed point, and realize the spatiotemporally controllable release of drugs [181]. For example, after MMP-2-containing PLGLAG sequencing responds to hydrogel-loading IL-4 or paclitaxel (PTX), it quickly degrades and releases drugs in the MMP-2 high-expression region, inhibiting the progression of melanoma and reshaping the local immune microenvironment [179]. In the load tumor model, MMP-2-sensitive GNE/nHA hydrogel can significantly inhibit the tumor, with lower tumor weight and slower growth (Figure 6D). After 12 days of treatment, the tumor volume was significantly suppressed, and the anti-tumor effect was remarkable (Figure 6E); the weight was stable during the experiment, which proved that its biocompatibility was good and the systemic toxicity was extremely low (Figure 6F) [179].

At present, a dual-responsive system has been developed. At the same time, hydrogels with dissolving bonds between MMP and ROS have been introduced, which can release AMPs through the dual triggering of enzymes and oxidation, improving release specificity and reducing cytotoxicity. AMP hydrogels GE33 can depolymerize in response to pH in an acidic stomach environment. First, active peptides are quickly released, and the residual network remains stable to achieve long-term residence and continuous drug effect [76]. In addition, short peptides such as those formed by the decapeptide KIKIPPIKIK can self-assemble to form a stable nanofiber structure, contain hydrophobic drugs such as paclitaxel, and achieve controlled release in the weak acidic environment of the tumor [16]. In vitro and in vivo experiments have confirmed that it has stronger anti-tumor effect and better safety, which can significantly reduce systemic drug exposure. In summary, protease response and multi-stimuli-responsive peptide hydrogels have good adaptability, which can realize the accurate delivery of drugs according to the pathology and treatment needs of the disease.

The outstanding advantage of peptide hydrogels is that they can load a variety of therapeutic drugs through non-covalent interactions such as hydrogen bonding, π–π stacking and hydrophobicity. Such a carrier can contain small molecule drugs, active peptides, proteins and nucleic acids, and accurately regulate the release rate and biological activity [182]. For example, enzyme-responsive self-assembled peptide hydrogels are applicable for eye administration to achieve localized sustained release of anti-inflammatory drugs and facilitate tissue repair [183]. GLP-1-derived peptide hydrogels can achieve the localized sustained release of polypeptide drugs [184]; the RADA16 scaffold loaded with dentin peptide can effectively promote vascular bone regeneration [143]. After being combined with nanocarriers, their function is further expanded, and the composite agent can improve the penetration and stability of the mucosa.

With its adjustable supramolecular structure, good biocompatibility and programmable molecular interaction, peptide hydrogels have become a multifunctional carrier in the field of nucleic acid delivery [12]. Its connected porous network and adjustable surface charge can stably bind siRNA, mRNA, CRISPR/Cas components and other nucleic acid drugs through electrostatic and hydrophobic effects [185]. This structure can reduce the early degradation and uncontrolled burst release of nucleic acids by nucleases, and improve the stability and bioavailability of nucleic acid drugs in lesion tissues. At the same time, the hydrogel hydration microenvironment can realize the continuous diffusion and local delivery of drugs, improve the accuracy of treatment and reduce systemic exposure.

At present, studies are further expanding the functionality of peptide hydrogels with photothermal, immune regulation or chemical modification systems, and optimizing the mechanical stiffness, degradation kinetics and nucleic acid loading capacity of materials. For example, β-peptide-based and chiral supramolecular hydrogels have been used for local gene delivery in ischemic injury models, which can achieve controlled release and site-specific transfection [186]. At the same time, the introduction of RALA and other cationic peptide derivatives into the hydrogel skeleton can improve nucleic acid binding and cell uptake efficiency, and provide a low-toxic carrier for local and systemic administration [187].

In summary, these advances show that peptide hydrogels have developed from a passive structural carrier to a multifunctional carrier that can realize long-term and spatially controlled gene delivery. Their modular structures and design flexibility are high, and they have broad application prospects in regenerative medicine, tumor treatment and precision genetic interventions for chronic inflammatory diseases.

### 5.3. Cell Therapy and Immunomodulation

In the field of cell therapy, functional peptide hydrogels can undertake structural support and immune regulation functions at the same time, with outstanding application potential. As a bionic three-dimensional ECM, they can provide spatial support and biological adhesion interfaces to promote cell adhesion, proliferation and directional differentiation. At the same time, by regulating the physicochemical characteristics such as local charge distribution and bioactive signaling motifs, the immune response is regulated to build a microenvironment conducive to cell survival and tissue repair [188]. Existing studies confirm that these hydrogels can improve the residence and functional stability of transplanted cells such as mesenchymal stem cells in the host tissue [189]. Their anti-inflammatory effect can also effectively improve the therapeutic efficacy of inflammatory bowel disease, intervertebral disk degeneration and other diseases [190].

The embedding of mesenchymal stem cells (MSCs) in polypeptide-modified hydrogels can construct a bionic three-dimensional microenvironment and effectively maintain cell activity and functional stability. The hydrogels are mostly prepared by microchannel or cryogelation to form a connected porous structure, which is conducive to the exchange of nutrients and oxygen and accelerates the discharge of metabolic waste. In addition, the adjustable degradation and programmed release characteristics of the matrix can realize the localized sustained release of bioactive molecules such as anti-inflammatory cytokines, exosomes and therapeutic ions [191].

In the early stage of wound repair, these hydrogels can promote the polarization of macrophages to M2 phenotypes, while inhibiting the secretion of inflammatory factors such as IL-1β, and enhance the anti-apoptotic activity and immune regulation of mesenchymal stem cells (MSCs) [192]. The study found that magnesium-rich hydrogels can induce M2 polarization and regulate osteogenic differentiation; hyaluronic acid scaffold modified by matrix cell-derived factor-1 (SDF-1) mimetic peptide can effectively recruit endogenous progenitor cells to the damaged area [191,193]. Similarly, hydrogels with gene-edited components or a decellularized extracellular matrix (dECM) can protect cartilage cells and promote the differentiation of bone marrow mesenchymal stem cells in the direction of cartilage.

Programmable peptide hydrogels modulate macrophage polarization via coordinated physicochemical properties and bioactive motifs with distinct molecular pathway specificity. Mechanical characteristics including network dynamics and 3D microstructure dominate immune regulation: enhanced network dynamics can significantly activate the JAK/STAT pathway to drive macrophage M2 polarization and facilitate granulation tissue formation and angiogenesis in diabetic wounds [194]. Combined with functional proteins such as decorin, the 3D microstructure suppresses the TLR4/MyD88/NF-κB cascade, thereby restraining persistent inflammation and fibrous capsule formation [195]. Mechanical cues also regulate the Hippo–YAP1 axis, which acts as a core mediator balancing M1/M2 phenotypes and linking macrophage metabolic reprogramming to polarization fate [196]. In parallel, specific peptide sequences enable pathway-selective immune tuning: certain peptide motifs trigger M1 polarization through NF-κB or TLR4 activation, while piezoelectric hydrogels remodel M1 toward M2 phenotypes via PI3K/Akt and TNF signaling inhibition [197]. Multiple downstream cascades including Rap1/PI3K/Akt, JAK2/STAT3, STAT6 and NOTCH pathways have also been validated to participate in hydrogel-mediated M2 polarization modulation [198,199,200,201].

Precise regulation of hydrogel stiffness and biochemical signals can further optimize cell behavior: a hard matrix can promote mesenchymal stem cell proliferation and YAP nuclear transposition, while a soft matrix is more conducive to immune regulation and paracrine signaling [202]. The introduction of adhesion sequences such as RGD can improve cell adhesion and spread; using dynamic covalent or supramolecular crosslinking, hydrogels are endowed self-healing, injectable and microenvironment responsiveness characteristics, so that they can fit irregular wounds and prolong retention time [6,203]. In addition, the multifunctional peptide hydrogels loaded with WR3-NH, DP7 and other AMPs and angiogenic domains can simultaneously achieve anti-infection, anti-inflammatory and neovascular tissue repair [190]. In summary, peptide hydrogels have both structural design, mechanical adjustability and biochemical functions. They can provide dynamic microenvironmental support for mesenchymal stem cells, significantly enhance their regenerative capacity in inflammation and diabetes wounds, and serve as a universal platform for the next generation of cell transplantation and precise regeneration therapies.

Despite the promising immunomodulatory outcomes, inappropriate material design may lead to immune imbalance and potential adverse risks. Abnormal M1 polarization induced by excessive NF-κB pathway activation may aggravate inflammatory damage and even exacerbate autoimmune pathological progression [204]. Improper degradation kinetics and unregulated immune modulation may break the inherent immune homeostasis, resulting in chronic local inflammation, ectopic fibrosis, and impaired tissue remodeling [205]. Long-term in vivo retention of hydrogel scaffolds may continuously interfere with macrophage dynamic balance, raising concerns regarding persistent immunogenicity and off-target inflammatory responses [206].

Peptide hydrogels have become an important multifunctional carrier for local immune regulation due to their excellent biocompatibility, programmable molecular structure and precise regulation of release kinetics. Their stable crosslinked three-dimensional network can efficiently contain a variety of immunologically active agents, realize spatial targeting and long-term release, mitigate systemic immune disturbances, and reduce non-targeted inflammatory reactions.

In tumor immunotherapy, elastin-like polypeptide (ELP) hydrogels are suitable as a dynamic delivery system to carry chemotherapy and immunotherapy drugs simultaneously. These injectable hydrogels can form in situ depots, containing gemcitabine and anti-PD-L1 antibodies, and achieving localized sustained release in the tumor microenvironment [207]. This strategy significantly enhances anti-tumor immune activation, manifested as increased cytotoxic CD8+ T cell infiltration, dendritic cell activation, and regulatory T cell reduction. These changes together reshape the immunosuppressive tumor microenvironment, enhance the systemic immune response, and improve the therapeutic effect of the preclinical model of melanoma.

In addition to the combination of chemotherapy and immunotherapy, the modular hydrogel vaccine platform can realize the local site-specific and spatiotemporally controlled delivery of tumor-related antigens and immunostimulants. For example, hydrogels equipped with CCL21a chemokine and tumor exosomes can recruit and activate dendritic cells, form long-term immune memory, and inhibit primary and metastatic tumor growth [208]. Similarly, hydrogels loaded with STING agonists and combined with photothermal treatment can effectively induce immunogenic cell death, promote the release of tumor antigen and damage-related molecular patterns, and enhance cytotoxic T cell response in cooperation with PD-1/PD-L1 blocking [209].

Hydrogel-mediated immune regulation has a significant advantage in local precise immune intervention. It can form clear immune effector microdomains in the lesion area, accurately regulate the tumor immune microenvironment, and reduce systemic immune-related adverse effects such as colitis and dermatitis. Their tunable three-dimensional networks can also recruit and retain immune cells such as dendritic cells, T cells, natural killer cells, etc., and promote the formation of tertiary lymph-like structures in tumors [210]. Through the above coordination mechanism, the hydrogel platform is expected to become an effective means for a new generation of local tumor treatment and postoperative immune intervention, which is used to mitigate the risk of tumor recurrence and metastasis.

Nevertheless, current immunomodulatory hydrogel strategies still face notable limitations and translational challenges. Tumor heterogeneity leads to highly variable macrophage polarization profiles regulated by intertwined NF-κB, STAT and NOTCH cascades, making single-pathway targeting difficult to achieve universal efficacy [100,211]. Severe physical barriers including high interstitial pressure and abnormal tumor vasculature greatly hinder the accumulation and functional execution of delivered hydrogel systems. Moreover, tumor cells can evade immune clearance through upregulated immune checkpoint pathways, while most existing hydrogel designs lack synergistic combinations with immune checkpoint blockade strategies. From a translational perspective, large-scale reproducible fabrication, consistent batch performance, long-term in vivo degradation controllability, and biosafety verification remain major bottlenecks [212,213]. In addition, achieving spatiotemporally precise perception and responsive regulation toward complex microenvironmental signals still requires further technical optimization [214].

### 5.4. Antibacterial and Antiviral Applications

The antimicrobial efficacy of functional peptide hydrogels primarily arises from the incorporation of bioactive peptide motifs capable of disrupting microbial membranes [215,216]. Such polypeptides are rich in cationic amino acids such as lysine and arginine, which can undergo strong electrostatic binding with the surface of negatively charged bacteria [12]. After binding, their hydrophobic segments penetrate the lipid double layer, destroying the integrity of the membrane and causing intracellular content leakage, and finally causing irreversible bacterial structural disruption and lysis. Recent studies have shown that peptide hydrogels can also exert antibacterial effects through other pathways, including producing reactive oxygen, interfering with key metabolic pathways, and triggering bacterial apoptosis in the cell [217]. Moreover, the hydrogel carrier can achieve the sustained release of active peptides, protect them from proteolytic degradation, and reduce the toxicity to mammalian cells [11]. This composite design reduces the generation of drug resistance while improving the therapeutic effect, and provides a feasible solution to deal with multidrug-resistant (MDR) bacteria such as Staphylococcus aureus and Pseudomonas aeruginosa.

In recent years, AMP hydrogels have garnered considerable attention as a multifunctional biological material for infection prevention and control and wound repair. The polysaccharide hydrogel loaded with AMPsWR3-NH (SGHC-WR) exhibits a porous microstructure , injectability, degradability, and a sustained polypeptide-release profile, which effectively inhibits pathogenic bacterial growth and accelerates the healing of infected wounds [218]. In another study, the pH-responsive co-assembled peptide hydrogels constructed by cationic short peptide Nap-FFKKK has obvious targeting of methicillin-resistant Staphylococcus aureus (MRSA) and can significantly accelerate wound healing [219]. In response to multidrug-resistant (MDR) bacterial infection, the hydrogel prepared by crosslinking oxidized dextran (ODEX) and the new polypeptide RWPIL by aldehyde can closely adhere to irregular wounds and effectively promote epithelial regeneration [220].

In terms of mechanisms of action, some AMP hydrogels can regulate the steady state of reactive oxygen species (ROS) and realize the synergy of antibacterial and immunomodulation [221]. The designed double peptide dynamic hydrogel has both antibacterial and angiogenesis-promoting functions; the hyaluronic acid hydrogel of complex MnO_2_ nanosheets improves the microenvironment of the infected wound through antibacterial and antioxidant synergy [222,223]. At present, studies mainly focus on the biocompatibility, stimulus responsiveness, controlled release kinetics and multifunctional synergy of peptide hydrogels, which lays a solid technical foundation for the development of clinical anti-infective wound dressings.

Compounding AMPs with the hydrogel matrix is an effective strategy to achieve synchronization between wound anti-infection and tissue regeneration. The introduction of DOPA-modified peptide segments (such as PonG1) has stronger interface stability and exerts significant bactericidal activity on Staphylococcus aureus (containing MRSA). Histological staining confirmed that this material can significantly accelerate wound healing and promote collagen deposition and angiogenesis in the full-layer infected wound model [188]. Similarly, the GelMA–dopamine complex hydrogel (GDHA) has excellent biocompatibility and mechanical properties, which can effectively promote wound repair [224].

For complex bacterial infections, a variety of complex hydrogel systems have been proven to have a synergistic therapeutic effect. Alginate/gelatin hydrogels loaded with peptide SALSP can accelerate granulation tissue formation and epithelial repair in the polymicrobial infection wound models [225]. The hyaluronic acid (HAD@AMP) of the AMP splantaricin 149 has strong antibacterial ability, which can reduce the formation of biofilm and accelerate the re-epithelialization of wounds [226]. Moreover, the dual-peptide dynamic hydrogel, which integrates bee venom-derived and angiogenic peptides, achieves the dual effect of antibacterial and angiogenesis through cooperative crosslinking [227]. Gradient-pH GelMA hydrogels can reduce the local pH of the wound, inhibit the growth of microorganisms, and promote the migration of fibroblasts and endothelial cells, thus regulating the wound microenvironment [228].

In the study of antiviral materials, the rational design of peptide sequences has become a common means of constructing local delivery platforms, which can simultaneously modulate infection-associated microenvironments and immune response at the same time [16]. The chemically modified peptide backbone can be spontaneously assembled into nanostructured hydrogel, which has adjustable hydrophilicity, good biocompatibility, and is sensitive to environmental stimuli such as pH [102]. These carriers can efficiently encapsulate and sustainably release anti-tumor drugs, immunomodulators and other biologically active substances, maintain local high drug concentration, extend the action time, and protect labile molecules from enzymatic decomposition [229,230].

The hydrogel system has outstanding potential in liver-related applications. The short peptide hydrogels verified by rheology can be injected, with stronger anti-tumor effect in vivo and excellent biological safety. Cellulose nanofiber (CNF) hydrogel has good biocompatibility and can play an antiviral role through the dual mechanism of “binding and blocking”. Negatively charged supramolecular peptide hydrogels (SAPHs) can promote HepG2 spheroid formation and maintain hepatocyte function, which is suitable for liver in vitro model construction [230]. Furthermore, engineering hydrogels such as DexNB-GelSH can mimic the physicochemical characteristics of natural liver tissue and provide material support for liver organ culture and liver targeted delivery [231]. The existing review has systematically confirmed that the hydrogel carriers have high drug-loading efficiency, can achieve responsive release and multifunctional delivery, and have clinical translation value in the treatment of hepatocellular carcinoma.

It should be clear that the application of peptide hydrogels in the treatment of hepatitis B virus (HBV) is not confined to the theoretical stage. Research data confirms that they have biological feasibility: a hexapeptide (Poly6) derived from HBV can induce the production of induced nitric oxide synthase-positive dendritic cells, and it exerts significant antitumor activity in mice through the type I interferon-dependent mechanism, reflecting the immunomodulation potential of viral-derived peptides [232]. In addition, the short peptide MOTS-c encoded by mitochondria has a variety of biological activities and is currently being studied as a candidate molecule for the diagnosis and treatment of HBV infection [233]. The monoclonal antibody against hepatitis B surface antigen (HBsAg) developed from the memory B cells of vaccinated individuals has entered the preclinical research stage of chronic hepatitis B and D, which proves the application value of peptide- and protein-based preparations in virus-specific targeted therapy.

Hydrogels can also be used as an antiviral delivery vector, such as the cellulose nanofilament (CNF) system using the “binding and blocking” mechanism, and the active peptide hydrogels scaffold used in tissue engineering and biomedicine [88,229]. Future research should focus on improving the targeting accuracy of hepatocytes, introducing virus-specific epitopes or immune response subsequences into self-assembly scaffolds, and enhancing the structural stability of hydrogels in pathological environments. Treatment verification needs to directly intervene in HBV-related molecular targets (such as cccDNA, HBsAg), and explore the synergistic combination strategy of hydrogel platform and existing therapies such as interferon, siRNA, and nucleotide analogs [229]. The existing research has gradually advanced from conceptual design to functional material development, laying an important foundation for the construction of a clinically transformable hydrogel-based antiviral system.

## 6. Challenges and Future Perspectives

Clinical translation based on peptide hydrogels is a gradual and multi-stage development path, covering the whole process from basic research and preclinical verification to final treatment and application. To translate innovative findings from the laboratory to clinical practice, it not only requires iterative optimization of their physicochemical properties and functional characteristics, but also requires strict verification of its stability, repeatability and biocompatibility under a variety of physiological conditions. In the early research stage, the research focus is mainly on functional optimization and elucidation of the mechanism of action; in contrast, in the transformation stage, the focus is shifted to establishing large-scale, standardized, and strictly controlled manufacturing processes, and at the same time, comprehensive pharmacokinetic, pharmacodynamic, and toxicological evaluations are carried out to ensure that it meets the requirements of clinical application [234].

Peptide hydrogels are assembled from endogenous amino acid units, which are different from synthetic polymer scaffolds. Their degradation products are short peptides or free amino acids, which can be directly metabolized by host cells and have excellent biocompatibility. However, key issues still need to be solved before they can be widely applied in clinical practice: first, to ensure stable and repeatable self-assembly in different individual patients; second, to achieve precise spatiotemporal regulation of the distribution position and release dynamics of the material during surgical implantation or interventional delivery.

From the regulatory level, peptide hydrogels are mostly classified as a medical device–drug combination product which have the dual functions of structural support and biological activity delivery. Therefore, their approval requires meeting the regulatory requirements of medical devices and drugs at the same time. A strict toxicological evaluation of the degradation products is required to ensure that they do not accumulate in vivo and do not interfere with normal metabolic processes. At the same time, the artificially designed polypeptide matrix needs to be comprehensively evaluated for immunogenicity and long-term post-administration monitoring to reduce adverse reactions and autoimmune-related risks [100]. The whole process of design and preparation should adhere to the principle of clinical controllability to ensure that biological functional components only play a role in the target tissue or pathological microenvironment.

The core challenge in clinical practice lies in the repeatability and long-term stability of the therapeutic effect. Individual differences in patients (local pH, enzyme activity, different immune levels) will significantly affect the self-assembly, degradation and drug release kinetics of hydrogel [12,235]. Non-biological factors such as the route of administration, the injection site and the temperature and humidity during the operation will also induce variability in therapeutic efficacy [236]. In immunomodulation applications, inaccurate targeting may destroy the immune homeostasis of normal tissues; excessive inhibition of inflammatory reactions will increase the risk of opportunistic infection [237]. In response to the above problems, the existing new technology adopts reversible functionalization modification, which can abrogate the biological activity of hydrogels on demand through exogenous stimulation when abnormal signals occur, so as to improve clinical safety [9].

Despite the promising preclinical progress, the clinical translation of programmable peptide hydrogels still faces multiple systematic bottlenecks. From the material perspective, inherent peptide instability, potential immunogenicity, off-target biological effects, and low oral bioavailability substantially restrict in vivo application. The introduction of bioactive structural domains may also interfere with intrinsic self-assembly behavior, leading to uncontrollable network structure and performance deviation [238,239]. From manufacturing and regulatory aspects, large-scale standardized production remains challenging due to complex preparation workflows, unclear industry supervision pathways, and conventional trial-and-error design paradigms that greatly limit research efficiency [240,241]. In addition, it remains difficult to simultaneously balance dynamic responsiveness, mechanical robustness, and diversified biological functions, while satisfying clinical strict requirements for batch repeatability and long-term biosafety. Emerging strategies such as cyclic peptide design, PLGA microsphere delivery systems, and dynamic hydrogel construction have been attempted to overcome these limitations, and the deep integration of artificial intelligence and multi-scale computational modeling is regarded as a core approach to accelerate clinical transformation [242,243].

Future research will focus on improving the stability and batch repeatability of hydrogels under real clinical conditions to ensure structural stability and controllable drug release kinetics [12]. In terms of improving biocompatibility, we will focus on rational optimization of peptide sequences, introduce immune escape mechanisms, adopt fine surface modifications, and reduce non-specific immune activation. At the same time, it is necessary to systematically investigate the degradation path, cell–matrix interactions and signal regulation of different tissue microenvironments. The current important development direction is to build an intelligent hydrogel system which can dynamically respond to physiological signals such as pH, enzyme activity and inflammatory factors, and realize the precise controlled release of therapeutic drugs [244]. Compounding peptide hydrogels with other materials can also enhance mechanical properties and expand functions [245]. The use of artificial intelligence for peptide sequence design and performance prediction is expected to accelerate the research, development, optimization, and clinical translation of a new generation of high-performance hydrogels [245].

Compounding peptide hydrogels with functional components has become an important hybrid material strategy for performance upgrading. Typical classification includes pure peptide assemblies, peptide hydrogels modified with nanoparticles or growth factors, and peptide-tailored polymer networks [88]. Multi-component co-assembly and path-dependent fabrication methods enable precise responsiveness to pH and temperature stimuli, as well as flexible regulation of cell behavior and mechanical performance [20]. In addition, synthetic peptide amphiphiles can co-assemble with natural proteins to construct biomimetic hybrid hydrogels, integrating supramolecular programmability and biological specificity for advanced tissue engineering scenarios [246].

Computational modeling has evolved into an indispensable theoretical tool for guiding peptide hydrogel design and preclinical verification. Multi-scale simulation frameworks covering force field optimization and atomic-level interaction analysis help elucidate the inherent structure–performance relationship of nanofiber networks [247]. Specially designed hydrogel models simulating hepatocellular carcinoma microenvironments can optimize irreversible electroporation parameters in silico, avoiding the ethical risks of excessive in vivo trials [248]. The integration of computational simulation, stimulus-responsive design and parameter optimization further forms a closed-loop “computation–material–clinic” paradigm, supporting the development of personalized tumor treatment strategies [249].

AI-assisted design is reshaping the traditional trial-and-error research mode of peptide hydrogels. Interactive experimental machine learning feedback systems have been applied to screen more than 160 tetrapeptide sequences, greatly improving the prediction accuracy of hydrogel formation capability and establishing a reproducible rational design framework [250]. Machine learning and deep learning algorithms are widely adopted for high-throughput sequence screening, multi-performance optimization, and automatic experimental design, accelerating the transition from empirical exploration to data-driven innovation [251,252]. Advanced computational methods such as contact parallel cascade selection molecular dynamics (cPaCS-MD) also provide precise simulation tools for peptide–protein interaction analysis and bioactive peptide optimization [253].

Although there are still problems in technology, supervision and clinical translation, peptide hydrogel still shows great application potential in many biomedical fields. In tissue engineering, it can mimic the core characteristics of natural ECMs and provide a suitable microenvironment for cell adhesion, proliferation and directional differentiation. In the drug delivery system, its programmable structure and stimuli-responsive performance realize the precise spatiotemporal regulation of therapeutic drugs and significantly reduce systemic adverse effects. In regenerative medicine, peptide hydrogels have both structural scaffold and local carrier functions, which can load biologically active factors or therapeutic cells to promote orderly tissue regeneration and functional repair. With the rapid development of materials science, molecular design and intelligent biological engineering, peptide hydrogels are expected to achieve a substantial clinical breakthrough and lay the foundation for building safer, more accurate and efficient next-generation therapeutic regimens.

## 7. Conclusions

Peptide hydrogels are a new generation of programmable bionic materials which enables structural construction through precise molecular sequence design and controllable self-assembly. Such systems have excellent biocompatibility, and at the same time the mechanical and structural properties exhibit a wide tunable range. With these inherent advantages, peptide hydrogels have great application prospects in tissue engineering, targeted drug delivery and regenerative medicine. In order to achieve dynamic remodeling in response to biological stimuli while maintaining structural stability, it is necessary to finely modulate physicochemical parameters such as viscoelasticity, porosity, degradation dynamics and mechanical stiffness. The optimized hydrogel matrix can mimic the complex microenvironment of natural ECMs, providing suitable conditions for cell adhesion, migration, proliferation and directional differentiation. This high adaptability makes peptide hydrogels a universal platform for regulating the fate of cells and promoting a new generation of biomedical applications.

Peptide hydrogels have dual synergistic functions in biomedical applications: they can not only support cell proliferation and orderly growth as a three-dimensional scaffold, but they also regulate the fate of cells as a biologically active interface by introducing a functionalized polypeptide matrix. After the reasonable introduction of biological function sequences, such materials can acquire properties of immune regulation, angiogenesis promotion, and antibacterial or induction differentiation, transforming from simple structural scaffolds to a dynamic treatment system that directly participates in tissue repair and regeneration. The synergy of molecular-level biological activity and macrostructural adaptability enables hydrogels to participate in the entire process of inflammatory regulation, tissue remodeling, and other repair processes, effectively improving the speed and quality of tissue regeneration.

In the field of drug delivery, peptide hydrogels represent precisely designed multi-stimuli-responsive systems which can achieve accurate and context-adapted controlled drug release. By optimizing the molecular sequence, crosslinking structure and environmental response characteristics, the material can accurately regulate drug release in the spatiotemporal dimension. This release regulation can not only enhance the local efficacy, but can also reduce systemic exposure and related adverse effects. Therefore, peptide hydrogels are especially suitable for chronic inflammation, intractable wounds, tumors and other complex diseases. In such diseases, continuous and site-focused administration is the key to optimizing the therapeutic effect.

Although there are still formidable challenges regarding fabrication stability, batch repeatability, regulatory approval, inherent immunogenicity, and uncontrolled in vivo performance, the rapid advances in multi-scale computational modeling, artificial intelligence-assisted sequence design, and hybrid material engineering are gradually breaking through these translational bottlenecks. Rational optimization of self-assembly mechanisms together with standardized manufacturing protocols has greatly enhanced the structural robustness and long-term functional reliability of programmable peptide hydrogels. In-depth clarification of material–tissue interactions, immune modulation mechanisms, and microenvironmental adaptability further promotes their transformation from laboratory experimental platforms to clinically validated therapeutic carriers. The cross-disciplinary integration of molecular engineering, computational simulation, and intelligent design fully highlights the inherent translational value of peptide hydrogels, which act as a critical bridge connecting fundamental biomaterial research and precision clinical therapy.

In summary, peptide hydrogels integrate structural biomimicry, tunable functionality and intrinsic biocompatibility, and are promising cutting-edge materials in the next generation of regenerative medicine and therapeutic applications. With the continuous integration of multidisciplinary technologies such as molecular design, nanoscale fabrication and computational modeling, such systems are gradually achieving high repeatability, precise regulation and stable and reliable clinical performance. These advances will provide key support for reshaping the precision medicine paradigm and promoting organizational reconstruction and the transformative development of regenerative medicine.

## Figures and Tables

**Table 2:** Functionalization strategies and biomedical applications of programmable peptide hydrogels.

Design Strategy	Incorporated Bioactive Motif	Biological/Physiological Effect	Targeted Application	Key Outcomes	Representative References
Covalent conjugation	Vascular-regenerative peptides	Enhanced endothelial cell adhesion, migration, and network formation	Vascularized tissue engineering	Improved vasculogenic efficacy validated in organ-on-chip and animal models; RNA-seq confirmed pro-angiogenic signaling	[94,95]
D-peptide stereo-complexation	D-enantiomeric peptides	Proteolytic stability, reduced immunogenicity, antibacterial/hemostatic activity	Wound dressing, anti-adhesion barriers	Superior in vitro/in vivo stability vs. L-peptide counterparts; minimal cytotoxicity with effective bacterial killing	[96]
Stimuli-responsive self-assembly	pH/enzyme-sensitive motifs	Controlled drug release, tunable degradation kinetics	Targeted drug delivery	Extended enzymatic resistance; pH-triggered sol–gel transition enabled sustained payload release with enhanced antitumor efficacy	[97]
Bioactive motif integration	RGD cell-adhesive sequence; lysine-rich antimicrobial peptides	Promoted cell proliferation; selective bacterial killing	3D cell culture, infected wound healing	High cytocompatibility; accelerated cutaneous regeneration via inflammation modulation and collagen deposition	[98]
Peptide-nanomaterial hybridization	Peptide-guided assembly of AuNPs; SQD surface functionalization	Photothermal-triggered release; NO generation; electrochemical sensing	Immunotherapy, biosensors, bioelectronics	Synergistic chemo-photothermal therapy; anti-inflammatory macrophage modulation; high-sensitivity wearable biosensing with mechanical stability	[19,99]
Immune-modulatory functionalization	Cytokine-mimetic or adjuvant peptides	Tunable macrophage polarization; controlled immune activation	Cancer immunotherapy, vaccine delivery	Demonstrated capacity to direct desired immune responses; reduced off-target cytotoxicity through motif optimization	[100]

**Table 3:** Porous characteristics and physical properties of peptide hydrogels fabrication methods.

Fabrication Method	Pore Size Range (μm/nm)	Network Connectivity	Mechanical Stiffness (kPa)	Biocompatibility	Main Applications	Limitations & Challenges	Representative References
Photopolymerization-induced phase separation	1–200 μm	Interconnected macroporous network	Tunable; elastic modulus varies by >10-fold with crosslinking density and irradiation time	High (peptide-derived, ECM-mimetic)	Tissue engineering scaffolds, 3D cell culture platforms	Stiffness-pore size interdependence requires precise parameter control; photoinitiator residues may affect cell viability	[125]
Chemical crosslinking (pyrrole-mediated)	15 μm	Uniform microporous structure (65% porosity)	15 (compressive modulus)	Excellent (in vitro cytocompatibility confirmed)	Antibacterial wound dressings, bioactive coatings	Compressive modulus limited to ~15 kPa; insufficient for load-bearing applications requiring >30 kPa	[126]
Self-assembly with Zn^2+^ coordination	1–100 μm	Nanofibrillar network (fibril diameter: 20–200 nm)	Enhanced stiffness via Zn complexation	High (supports cell adhesion/function)	Antibacterial/anti-inflammatory platforms, regenerative matrices	Hierarchical structure reproducibility challenging; mechanical reinforcement dependent on Zn concentration	[127]
Ice crystal-assisted templating	50–200 μm	Spongy, highly interconnected macroporous network	Adequate for soft tissue support	High (chitosan/ε-polylysine-based system)	Infected burn wound management, nutrient/gas exchange media	Pore size > 100 μm correlates with reduced structural integrity; compressive modulus typically <10 kPa in large pore configurations	[128]
Supramolecular self-assembly (ultra-short peptides)	Nanoscale network pores	Nanofibrillar network (fibril diameter: 20–200 nm)	Typically 0.1–10 kPa	Excellent (low immunogenicity, biodegradable)	Immune modulation, drug delivery, 3D bioprinting bioinks	Compressive modulus frequently <5 kPa without reinforcement; limited shape fidelity under physiological stress	[91]

## Data Availability

No new data were created or analyzed in this study. Data sharing is not applicable to this article.

## Abbreviations

The following abbreviations are used in this manuscript:

3D	Three-Dimensional
AKT	Protein Kinase B
ALP	Alkaline Phosphatase
AMP	Antimicrobial Peptide
Arg-1	Arginase 1
AuNPs	Gold Nanoparticles
BSAMA	Bovine Serum Albumin Methacryloyl
BMP2	Bone Morphogenetic Protein 2
BMSC	Bone Marrow Mesenchymal Stem Cell
β-catenin	Beta-Catenin
CGRP	Calcitonin Gene-Related Peptide
CCL21a	C-C Motif Chemokine Ligand 21a
cRGD	Cyclic Arginine-Glycine-Aspartic Acid
CNF	Cellulose Nanofiber
CM	Chondrocyte Matrix
CMKGN	Chondrocyte Matrix Kartogenin
CNS	Central Nervous System
CRISPR/Cas	Clustered Regularly Interspaced Short Palindromic Repeats/CRISPR-Associated Protein
CT	Computed Tomography
DAPI	4’,6-Diamidino-2-Phenylindole
Dentonin	Functional Peptide Dentonin
DGL	Dendritic Polylysine
DOPA	Dopamine
DP7	Antimicrobial Peptide DP7
dECM	Decellularized Extracellular Matrix
ECM	Extracellular Matrix
EDC	1-Ethyl-3-(3-dimethylaminopropyl) Carbodiimide
EGFR	Epidermal Growth Factor Receptor
ELP	Elastin-Like Polypeptide
EMT	Epithelial–Mesenchymal Transition
Fmoc-γPhe-Phe-OH	Ultrashort Hybrid Peptide
FEFK	Peptide Sequence
FKFE	Peptide Sequence
FFFGHK	Peptide Sequence FFFGHK
G7.5	Gelatin Methacryloyl 7.5
GA	Glycyrrhizic Acid
GAG	Glycosaminoglycan
GelMA	Gelatin Methacryloyl
GE33	Antimicrobial Peptide Hydrogel GE33
GFAP	Glial Fibrillary Acidic Protein
GFOGER	Peptide Sequence
GGQQLK	Peptide Sequence
GHK	Gly-His-Lys
GLP1	Glucagon-Like Peptide 1
GNE	Glycomacropeptide
H&E	Hematoxylin and Eosin
HA	Hyaluronic Acid
HBD	vWF-Derived Peptide
HBV	Hepatitis B Virus
HBsAg	Hepatitis B Surface Antigen
IκBα	Nuclear Factor of Kappa Light Polypeptide Gene Enhancer in B-Cells Inhibitor Alpha
IKVAV	Ile-Lys-Val-Ala-Val
IL-1β	Interleukin 1 Beta
IL-4	Interleukin 4
IL-10	Interleukin 10
iNOS	Inducible Nitric Oxide Synthase
iPSC	Induced Pluripotent Stem Cell
KGN	Kartogenin
KIKIPPIKIK	Decapeptide Sequence
LAP	Laponite
MAX1	β-Hairpin Peptide MAX1
MAP2	Microtubule-Associated Protein 2
MHC	Myosin Heavy Chain
MMP	Matrix Metalloproteinase
MMP-2	Matrix Metalloproteinase 2
MMP-9	Matrix Metalloproteinase 9
MMP13	Matrix Metalloproteinase 13
nHA	Nano-Hydroxyapatite
NF-κB	Nuclear Factor Kappa B
NGF	Nerve Growth Factor
NHS	N-Hydroxysuccinimide
PAE3Y	Peptide Amphiphile E3Y
PCL	Polycaprolactone
PEG	Polyethylene Glycol
PD-1	Programmed Cell Death Protein 1
PD-L1	Programmed Death-Ligand 1
PDOXAP	Prodrug Oxaliplatin Apoptin
PI3K	Phosphatidylinositol 3-Kinase
PLGLAG	Peptide Sequence PLGLAG
PTX	Paclitaxel
Q	Laminin-Derived Q Peptide
QGT-IK	Peptide Sequence
QHREDGS	Peptide Sequence
RADA16	Self-Assembling Peptide RADA16
RAD	Self-Assembling Peptide RAD
RALA	Cationic Peptide RALA
RKRLQVQLSIRTC	Peptide Sequence
RGD	Arg-Gly-Asp
ROS	Reactive Oxygen Species
Runx2	Runt-Related Transcription Factor 2
SA	Sodium Alginate
SAPH	Supramolecular Peptide Hydrogel
siRNA	Small Interfering RNA
SDF1	Stromal Cell-Derived Factor 1
STING	Stimulator of Interferon Gene
TGF-β1	Transforming Growth Factor Beta 1
TNF-α	Tumor Necrosis Factor Alpha
Treg	Regulatory T Cell
TYRAY	Vascular-Regenerative Peptide
VEGF	Vascular Endothelial Growth Factor
vWF	Von Willebrand Factor
PVA	Polyvinyl Alcohol
WR3NH	Antimicrobial Peptide WR3NH
YIGSR	Tyr-Ile-Gly-Ser-Arg
MRSA	Methicillin-Resistant Staphylococcus Aureus
PdBT	Peptide Sequence PdBT
CU	Cryptotanshinone–Peptide

## References

[1] Li H., Murugesan A., Shoaib M., Chen Q. (2025). Emerging Trends and Future Prospects of Peptide-Based Hydrogels: Revolutionizing Food Technology Applications. Compr. Rev. Food Sci. Food Saf..

[2] Kwon W.H., Choi K., Park S.J., Park G., Park C.Y., Seo Y.H., Kim C.H., Choi J.S. (2025). Enhancing the Biological Functionality of Hydrogels Using Self-Assembling Peptides. Biomimetics.

[3] Lowen J.M., Wheeler E.E., Shimamoto N.K., Ramos-Rodriguez D.H., Griffin K.H., Bond G.C., Leach J.K. (2024). Functionalized Annealed Microgels for Spatial Control of Osteogenic and Chondrogenic Differentiation. Adv. Funct. Mater..

[4] Liu P.L., He S.H., Shen Z.H., Li X.R., Deng Q.S., Wei Z.Y., Zhang C.R., Dou X.Q., Zhu T.H., Dawes H. (2025). Bilayer Scaffolds Synergize Immunomodulation and Rejuvenation via Layer-Specific Release of CK2.1 and the “Exercise Hormone” Lac-Phe for Enhanced Osteochondral Regeneration. Adv. Healthc. Mater..

[5] Mohanty S., Roy S. (2025). Fabricating N-Cadherin Mimetic Peptide-Based Diverse Self-Assembled Hydrogels in the Presence of Biologically Relevant Cations. Biomacromolecules.

[6] Mathes T.G., Kim U., Jeon K., Estevez P.J., Terasaki M., Ermis M., O’Raw A., Jucaud V., Khademhosseini A., Falcone N. (2025). Lipopeptide Hydrogel Possesses Adjuvant-like Properties for the Delivery of the GPC-3 Peptide-derived Antigen. Adv. Funct. Mater..

[7] Wu C., Liao W., Zhang Y., Yan Y. (2024). Peptide-based supramolecular hydrogels and their biotherapeutic applications. Biomater. Sci..

[8] Noddeland H.K., Lind M., Jensen L.B., Petersson K., Skak-Nielsen T., Larsen F.H., Malmsten M., Heinz A. (2023). Design and characterization of matrix metalloproteinase-responsive hydrogels for the treatment of inflammatory skin diseases. Acta Biomater..

[9] Zhou H., Zhu Y., Yang B., Huo Y., Yin Y., Jiang X., Ji W. (2024). Stimuli-responsive peptide hydrogels for biomedical applications. J. Mater. Chem. B.

[10] Zhao J., Wu S., Zhang M., Hong X., Zhao M., Xu S., Ji J., Ren K., Fu G., Fu J. (2024). Adventitial delivery of miR-145 to treat intimal hyperplasia post vascular injuries through injectable and in-situ self-assembling peptide hydrogels. Acta Biomater..

[11] Qu H., Yao Q., Chen T., Wu H., Liu Y., Wang C., Dong A. (2024). Current status of development and biomedical applications of peptide-based antimicrobial hydrogels. Adv. Colloid Interface Sci..

[12] Asokan-Sheeja H., Awad K., Xu J., Le M., Nguyen J.N., Nguyen N., Nguyen T.P., Nguyen K.T., Hong Y., Varanasi V.G. (2024). In Situ Synthesis and Self-Assembly of Peptide-PEG Conjugates: A Facile Method for the Construction of Fibrous Hydrogels. Biomacromolecules.

[13] Guerrero M., Filho D., Ayala A.N., Rafael D., Andrade F., Marican A., Vijayakumar S., Duran-Lara E.F. (2025). Hydrogel-antimicrobial peptide association: A novel and promising strategy to combat resistant infections. Colloids Surf. B Biointerfaces.

[14] Dong S., Han Y., Wang Y., Ding Q., Ding C., Chen S., Song Y., Zhao T. (2025). Multifunctional hydrogel scaffolds loaded with peptides in promoting wound healing: A review. Int. J. Biol. Macromol..

[15] Perin F., Ricci A., Fagiolino S., Rak-Raszewska A., Kearney H., Ramis J., Bereen I., Baker M., Maniglio D., Motta A. (2025). Bioprinting of Alginate-Norbornene bioinks to create a versatile platform for kidney in vitro modeling. Bioact. Mater..

[16] Yao Q., Gao J., Liu L., Shi J., Zafar H., Khan M.I., Zhu J., Raza F., Zhu Y. (2025). Short peptide hydrogel with angular structure for hydrophobic antitumor drug delivery and controlled release. Colloids Surf. B Biointerfaces.

[17] Duti I.J., Paul J., Reilly K.S., Miller D.R., Dickie D.A., Letteri R.A. (2025). Peptide stereocomplex cross-links for polymer hydrogels. Chem. Sci..

[18] Zhu C., Wu W., Soladoye O.P., Zhang N., Zhang Y., Fu Y. (2024). Towards food-derived self-assembling peptide-based hydrogels: Insights into preparation, characterization and mechanism. Food Chem..

[19] Younis M., Tabish T.A., Firdharini C., Aslam M., Khair M., Anjum D.H., Yan X., Abbas M. (2025). Self-Assembled Peptide-Based Fibrous Hydrogel as a Biological Catalytic Scaffold for Nitric Oxide Generation and Encapsulation. ACS Appl. Mater. Interfaces.

[20] Mohanty S., Sen S., Sharma P., Roy S. (2024). Designing Pathway-Controlled Multicomponent Ultrashort Peptide Hydrogels with Diverse Functionalities at the Nanoscale for Directing Cellular Behavior. Biomacromolecules.

[21] Wang K.H., Liu C.H., Tan D.H., Nieh M.P., Su W.F. (2024). Block Sequence Effects on the Self-Assembly Behaviors of Polypeptide-Based Penta-Block Copolymer Hydrogels. ACS Appl. Mater. Interfaces.

[22] Brownell D., Carignan L., Alavi R., Caneparo C., Labroy M., Galbraith T., Chabaud S., Berthod F., Gibot L., Bordeleau F. (2025). Impact of the Use of 2-Phospho-L Ascorbic Acid in the Production of Engineered Stromal Tissue for Regenerative Medicine. Cells.

[23] Chen C., Wu D., Wang Z., Liu L., He J., Li J., Chu B., Wang S., Yu B., Liu W. (2024). Peptide-Based Hydrogel Scaffold Facilitates Articular Cartilage Damage Repair. ACS Appl. Mater. Interfaces.

[24] Loth C., Barbault F., Guegan C., Lemaire F., Contal C., Carvalho A., Helle S., Champion M., Kerdjoudj H., Chan-Seng D. (2025). Experimental and Computational Study of Injectable Iron(III)/Ultrashort Peptide Hydrogels: A Candidate for Ferroptosis-Induced Treatment of Bacterial Infections. Small Sci..

[25] Nasiru M.M., Boateng E.F., Alnadari F., Bako H.K., Ibeogu H.I., Feng J., Song J., Liu H., Zhang Q., Masisi K. (2025). Cold plasma reengineers peanut protein isolate: Unveiling changes in functionality, rheology, and structure. Int. J. Biol. Macromol..

[26] Samdin T.D., Lubkowski J., Anderson C.F., Schneider J.P. (2025). From Hydrogel to Crystal: A Molecular Design Strategy that Chemically Modifies Racemic Gel-Forming Peptides to Furnish Crystalline Fibrils Stabilized by Parallel Rippled beta-Sheets. J. Am. Chem. Soc..

[27] Bertouille J., White J.F., de Vries M., De Smet K., Zhang J., Gardiner J., Van den Brande N., Herrebout W., Willaert R.G., Martin C. (2025). Influence of substituted aromatics on the formation and stability of beta-sheet-based peptide hydrogels. Nanoscale.

[28] Zhao S., Xue C., Burns D.C., Shoichet M.S. (2024). Viscoelastic Supramolecular Hyaluronan-Peptide Cross-Linked Hydrogels. Biomacromolecules.

[29] Nadimifar M., Jin W., Coll-Satue C., Bor G., Kempen P.J., Moosavi-Movahedi A.A., Hosta-Rigau L. (2024). Synthesis of bioactive hemoglobin-based oxygen carrier nanoparticles via metal-phenolic complexation. Biomater. Adv..

[30] Zoghi N., Yang C.Y., Bryce R., Miller A.F., Saiani A. (2025). Effect of C-Terminal Residue on the Phase Behavior and Properties of beta-Sheet Forming Self-Assembling Peptide Hydrogels. Biomacromolecules.

[31] Bunuasunthon S., Nakamoto M., Hoven V.P., Matsusaki M. (2024). Construction of Tough Hydrogel Cross-Linked via Ionic Interaction by Protection Effect of Hydrophobic Domains. ACS Biomater. Sci. Eng..

[32] Ge L., Xu B. (2025). Self-Healing Fire Prevention and Extinguishing Hydrogel Derived from Carboxymethyl Cellulose-Modified Amphiphilic Copolymers. Gels.

[33] Cheng Q., Hao A., Xing P. (2024). Engineering pi-Conjugation of Phenylalanine Derivatives for Controllable Chiral Folding and Self-Assemblies. ACS Nano.

[34] Chen Z., Guo C., Wang L., Chen C., Cai J., Liu C., Gan Z., Sun Y., Zhou J., Zhou J. (2024). Electrostatic Potential Design of Solid Additives for Enhanced Molecular Order of Polymer Donor in Efficient Organic Solar Cells. Small.

[35] Zhao P., Zhao Y., Lu Y., Xu L., Li B., Zhao Y., Zhou W., Yan P., Wang Y., Cao K. (2024). Non-Equilibrium Dissipative Assembly with Switchable Biological Functions. Angew. Chem. Int. Ed. Engl..

[36] Soliman M.A.N., Khedr A., Sahota T., Armitage R., Allan R., Laird K., Allcock N., Ghuloum F.I., Amer M.H., Alazragi R. (2025). Unraveling the Atomistic Mechanism of Electrostatic Lateral Association of Peptide beta-Sheet Structures and Its Role in Nanofiber Growth and Hydrogelation. Small.

[37] Criado-Gonzalez M., Penas M.I., Barbault F., Muller A.J., Boulmedais F., Hernandez R. (2024). Salt-induced Fmoc-tripeptide supramolecular hydrogels: A combined experimental and computational study of the self-assembly. Nanoscale.

[38] Gopalakrishnan A., Janardhanan D.V., Sasi S., Aravindakumar C.T., Aravind U.K. (2024). Organic micropollutant removal and phosphate recovery by polyelectrolyte multilayer membranes: Impact of buildup interactions. Chemosphere.

[39] Bao L., Kang W.B., Zhu B.C., Xiao Y. (2025). Charge Arrangement Determines the Sensitivity of Aggregation Patterns between Peptide-Chains to the Surrounding Ionic Environment. J. Chem. Inf. Model..

[40] Cheng L., De Leon-Rodriguez L.M., Gilbert E.P., Loo T., Petters L., Yang Z. (2024). Self-assembly and hydrogelation of a potential bioactive peptide derived from quinoa proteins. Int. J. Biol. Macromol..

[41] Das T.N., Ramesh A., Ghosh A., Moyra S., Maji T.K., Ghosh G. (2025). Peptide-based nanomaterials and their diverse applications. Nanoscale Horiz..

[42] Wang X., Wang N., Zhou P., Guo R., Song N., Zhang Z., Yan X., Yu Z., Li G. (2025). Oligomerization Propensity Governs Self-Assembly Morphology: Insights from Steady-State Oligomer Analysis. Anal. Chem..

[43] Yu S., Ye Z., Roy R., Sonani R.R., Pramudya I., Xian S., Xiang Y., Liu G., Flores B., Nativ-Roth E. (2024). Glucose-Triggered Gelation of Supramolecular Peptide Nanocoils with Glucose-Binding Motifs. Adv. Mater..

[44] Ma X., Qi K., Ju X., Sun Y., Yang H., Ke Y., Zhang J., Zhao Y., Xu H., Wang J. (2025). pH-Dependent Packing Mode Variations and Chirality Inversion in Short Peptide Self-Assembly. Angew. Chem. Int. Ed. Engl..

[45] Luo Q., Luo J., Luan Z., Xu K., Tian L., Zhang K., Peng X., Yuan M., Zheng C., Shu Z. (2024). Blue Laser Triggered Hemostatic Peptide Hydrogel for Gastrointestinal Bleeding Treatment. Adv. Mater..

[46] Feng Y., Zhang Z., Yan W., Zhang Z., Li Y., Zhu C., Fan D. (2025). A Sprayable Hydrogel Based on Biomimetic Polypeptide-Modified Lipid Nanoparticles for Treating Non-Compressible Hemorrhaging. Adv. Mater..

[47] Meisenhelter J.E., Petrich N.R., Blum J.E., Weisen A.R., Guo R., Saven J.G., Pochan D.J., Kloxin C.J. (2024). Impact of Peptide Length and Solution Conditions on Tetrameric Coiled Coil Formation. Biomacromolecules.

[48] Wei G., Zong B., He Q., Su S., Li Y., Zheng J., Qian Y., Cao P., Li Z. (2024). A Thin Polymer Layer Enables Peptide-Polycation Complexes with Ultrahigh Efficient Encapsulation. Small.

[49] Xu Z., Han S., Guan S., Zhang R., Chen H., Zhang L., Han L., Tan Z., Du M., Li T. (2024). Preparation, design, identification and application of self-assembly peptides from seafood: A review. Food Chem. X.

[50] Ma T., Yu Y., Gao Y., Jiang S., Ge W., Zeng Y., Wang X., Li S., Xie X., Guan G. (2025). Smart self-assembled peptide-based hydrogels: Mechanism, design and biomedical applications. Colloids Surf. B Biointerfaces.

[51] Hou M., Liu S. (2024). Recent Progress of pH-Responsive Peptides, Polypeptides, and Their Supramolecular Assemblies for Biomedical Applications. Biomacromolecules.

[52] Liu H., Henderson M., Pang Z., Zhang Q., Lam E., Liu Q. (2025). Structural determinants for pH-dependent activation of a plant metacaspase. Nat. Commun..

[53] Li J., Li Y., Koide A., Kuang H., Torres V.J., Koide S., Wang D.N., Traaseth N.J. (2024). Proton-coupled transport mechanism of the efflux pump NorA. Nat. Commun..

[54] Guo K., Gao L., Fei J. (2025). Cascade of phase transitions in a dipeptide supramolecular assembly triggered by a single fatty acid. Colloids Surf. B Biointerfaces.

[55] Tapponi S., Yusuf A., Alsaafin F., Hussain Z. (2025). Breaking barriers with pH-responsive nanocarriers: A new frontier in precision oncology. Int. J. Pharm..

[56] Mei H., Huang Y., Yi J., Chen W., Guan P., Guan S., Chen X., Li W., Yang L. (2025). Molecular Dynamics Simulation of the Thermosensitive Gelation Mechanism of Phosphorylcholine Groups-Conjugated Methylcellulose Hydrogel. Gels.

[57] Miyaguni I., Homma K., Matsusaki M. (2025). Incorporation of Visible Light-Responsive Push-Pull Azobenzene into Polymer Networks toward the Construction of Photodynamic Hydrogel Scaffolds. ACS Macro Lett..

[58] Xiao Z., Liu J., Song Z., Du T., Du X. (2025). Advances in hydrogels combined with photothermal/photodynamic therapy for bacterial infection. J. Mater. Chem. B.

[59] Zhang Q., Hu W., Guo M., Zhang X., Zhang Q., Peng F., Yan L., Hu Z., Tangthianchaichana J., Shen Y. (2024). MMP-2 Responsive Peptide Hydrogel-Based Nanoplatform for Multimodal Tumor Therapy. Int. J. Nanomed..

[60] Chen W., Sheng S., Tan K., Wang S., Wu X., Yang J., Hu Y., Cao L., Xu K., Zhou F. (2024). Injectable hydrogels for bone regeneration with tunable degradability via peptide chirality modification. Mater. Horiz..

[61] Tang J., Hao M., Liu J., Chen Y., Wufuer G., Zhu J., Zhang X., Zheng T., Fang M., Zhang S. (2024). Design of a recombinant asparaginyl ligase for site-specific modification using efficient recognition and nucleophile motifs. Commun. Chem..

[62] Wang G., Lu T., Zhang L., Ding J. (2024). IRF2BP2 binds to a conserved RxSVI motif of protein partners and regulates megakaryocytic differentiation. Nat. Commun..

[63] Pareek A., Bhatt B., Parmar V., Alasiri G., Alsaidan O.A., Kapoor D.U., Prajapati B.G. (2025). pH-Sensitive hydrogels for breast cancer therapy: Targeted drug delivery and controlled release approaches. Int. J. Pharm..

[64] Gentile L., Frohm B., Malmendal A., Akerfeldt K.S., Olsson U., Linse S. (2025). Charge regulation in peptide self-assembly and hydrogelation. J. Colloid Interface Sci..

[65] Xia R., Sun W., Liang J., Wang S., Gao F., Liu Y., Wang L., Zhang H., Ma C., Liu K. (2025). Few-Shot Identification of Active Therapeutic Peptide Networking with Gradient Dynamics against Cancer. Adv. Healthc. Mater..

[66] Rosa E., Pizzella M., Cimmino L., Castelletto V., Hamley I.W., Vitagliano L., De Simone A., Accardo A. (2025). Stimuli-Responsive Hydrogels from Liquid-Liquid Phase Separations of FUS-Derived Peptides. ACS Appl. Mater. Interfaces.

[67] Adorinni S., Kurbasic M., Garcia A.M., Kralj S., Bellotto O., Scarel E., Pengo P., De Zorzi R., Melchionna M., Vargiu A.V. (2024). A water playground for peptide re-assembly from fibrils to plates. J. Mater. Chem. B.

[68] Lv T., Chen Y., Li N., Liao X., Heng Y., Guo Y., Hu K. (2025). A Comprehensive Review of Thermosensitive Hydrogels: Mechanism, Optimization Strategies, and Applications. Gels.

[69] Xiang Y., Mao H., Tong S.C., Liu C., Yan R., Zhao L., Zhu L., Bao C. (2023). A Facile and Versatile Approach to Construct Photoactivated Peptide Hydrogels by Regulating Electrostatic Repulsion. ACS Nano.

[70] Wang Y.Y., Chen P.W., Chen Y.H., Yeh M.Y. (2025). Research on advanced photoresponsive azobenzene hydrogels with push-pull electronic effects: A breakthrough in photoswitchable adhesive technologies. Mater. Horiz..

[71] Li C., Wu Y., Bao S., Li H., Xu Z., Yan J., Yu X., He L., Zhang T., Liu W. (2025). Photo-Switchable Supramolecular Interactions Regulate K(+) Transmembrane Transport and Cancer Cell Apoptosis. J. Am. Chem. Soc..

[72] Wu M., Liu H., Li D., Zhu Y., Wu P., Chen Z., Chen F., Chen Y., Deng Z., Cai L. (2024). Smart-Responsive Multifunctional Therapeutic System for Improved Regenerative Microenvironment and Accelerated Bone Regeneration via Mild Photothermal Therapy. Adv. Sci..

[73] Zhu Y., Liu H., Wu P., Chen Y., Deng Z., Cai L., Wu M. (2024). Multifunctional injectable hydrogel system as a mild photothermal-assisted therapeutic platform for programmed regulation of inflammation and osteo-microenvironment for enhanced healing of diabetic bone defects in situ. Theranostics.

[74] Lu Y., Li S., Deng F., Yue Y., He S., Jiang S., Ye M., Zhou Y., Xiao H., Han J. (2026). Programmable and multi-stimuli responsive hydrogel actuator mediated by nanocellulose with intrinsic self-sensing capacity. Carbohydr. Polym..

[75] Liu L., Wang W., Huang L., Xian Y., Ma W., Zhao L., Li Y., Zheng Z., Liu H., Wu D. (2024). Injectable Inflammation-Responsive Hydrogels for Microenvironmental Regulation of Intervertebral Disc Degeneration. Adv. Healthc. Mater..

[76] Guo Z., Hou Y., Tian Y., Tian J., Hu J., Zhang Y. (2024). Antimicrobial Peptide Hydrogel with pH-Responsive and Controllable Drug Release Properties for the Efficient Treatment of Helicobacter pylori Infection. ACS Appl. Mater. Interfaces.

[77] Lu P., Shan M., Peng C., Ji W., Yang T., Yang Z., Zhang Z., Wang Y. (2025). Alkaline Phosphatase-Triggered Spatiotemporal Repair of Corneal Injury with TB500 Peptide Hydrogel. ACS Appl. Mater. Interfaces.

[78] Chen Q., Liang Z., Wang W., Wang N., Zhang K., Liu G., Li M. (2025). A pH and Light Dual-Responsive Spiropyran-Peptide Self-Assembly for Controlled Mitochondria and Nucleus Targeting. Nano Lett..

[79] Suryavanshi P., Mahajan S., Banerjee S.K., Seth K., Banerjee S. (2024). Synthesis and characterization of a pH/temperature-dual responsive hydrogel with promising biocompatibility features for stimuli-responsive 5-FU delivery. J. Mater. Chem. B.

[80] Bigo-Simon A., Estrozi L.F., Chaumont A., Schurhammer R., Schoehn G., Combet J., Schmutz M., Schaaf P., Jierry L. (2024). 3D Cryo-Electron Microscopy Reveals the Structure of a 3-Fluorenylmethyloxycarbonyl Zipper Motif Ensuring the Self-Assembly of Tripeptide Nanofibers. ACS Nano.

[81] Jain M., Trapani G., Trappmann B., Ravoo B.J. (2024). Stiffness Modulation and Pulsatile Release in Dual Responsive Hydrogels. Angew. Chem. Int. Ed. Engl..

[82] Quan L., Xin Y., Zhang Z., Zhou C., Ao Q. (2025). Peptide-Enhanced Bioactive Hydrogel Combined with Photodynamic-Phage Synergistic Antibacterial Therapy System Accelerates Infected Wound Healing. Adv. Healthc. Mater..

[83] Li S., Li X., Xu Y., Fan C., Li Z.A., Zheng L., Luo B., Li Z.P., Lin B., Zha Z.G. (2024). Collagen fibril-like injectable hydrogels from self-assembled nanoparticles for promoting wound healing. Bioact. Mater..

[84] Nie X., Hui J., Han Z., Wang H., Zhou Y., Shao J., Wang L., Xu Z., Wu B., Cui C. (2026). A natural corneal extracellular matrix-inspired dual-crosslinked hydrogel bioadhesive for emergency corneal trauma repair. Acta Biomater..

[85] Hou Y., Li F., Liu W., Guo R., Wu H., Huang S., Xu C., Zhu L., Zhang J., Wei B. (2024). Unraveling the role of integrating signal peptides into natural collagen on modulating cancer cell adhesion. Int. J. Biol. Macromol..

[86] Hao R., Niu X., Jiang X., Liu K., Ma X., Chen C. (2025). Transglutaminase-triggered dual gradients of mechanical and biochemical cues self-assembling peptide hydrogel for guiding MC3T3-E1 cell behaviors. Int. J. Biol. Macromol..

[87] Qian X., Guan L., Shen L., Zhai C., Cheng Y., Pan G., Jiang Z. (2025). Recent advances in hydrogel-assisted treatment of malignant bone tumors. Mater. Today Bio.

[88] Sun Z., Hu H., Zhang X., Luan X., Xi Y., Wei G., Zhang X. (2024). Recent advances in peptide-based bioactive hydrogels for nerve repair and regeneration: From material design to fabrication, functional tailoring and applications. J. Mater. Chem. B.

[89] Hao Y., Yan J., Fraser C., Jiang A., Anuganti M., Zhang R., Lloyd K., Jardine J., Coppola J., Meijers R. (2024). Synthetic integrin antibodies discovered by yeast display reveal alphaV subunit pairing preferences with beta subunits. MAbs.

[90] Gonzalez-Perez F., Alonso M., Gonzalez de Torre I., Santos M., Rodriguez-Cabello J.C. (2022). Protease-Sensitive, VEGF-Mimetic Peptide, and IKVAV Laminin-Derived Peptide Sequences within Elastin-Like Recombinamer Scaffolds Provide Spatiotemporally Synchronized Guidance of Angiogenesis and Neurogenesis. Adv. Healthc. Mater..

[91] Sahu I., Chakraborty P. (2024). A repertoire of nanoengineered short peptide-based hydrogels and their applications in biotechnology. Colloids Surf. B Biointerfaces.

[92] Lou J., Meyer C., Chen A., Weitz D.A., Mooney D.J. (2025). Immobilization of BMP-2 in porous hydrogels to spatially regulate osteogenesis. J. Control. Release.

[93] Huang W., Guo Q., Wu H., Zheng Y., Xiang T., Zhou S. (2025). Engineered Exosomes Loaded in Intrinsic Immunomodulatory Hydrogels with Promoting Angiogenesis for Programmed Therapy of Diabetic Wounds. ACS Nano.

[94] Xing T., Wang X., Xu Y., Sun F., Chen M., Yan Q., Ma Z., Jiang H., Chen X., Li X. (2024). Click method preserves but EDC method compromises the therapeutic activities of the peptide-activated hydrogels for critical ischemic vessel regeneration. Biomed. Pharmacother..

[95] Chochola V., Spustova K., Lavicky J., Golunova A., Pospisil J., Dvorakova J., Kotelnikov I., Kandra M., Streit L., Szklanny A. (2025). TYRAY-Functionalized Alginate Bioinks for 3D Bioprinting Support Stem Cell Culture and Endothelial Network Formation. ACS Biomater. Sci. Eng..

[96] Zhang H., Wu Z., Zhou J., Wang Z., Yang C., Wang P., Fareed M.S., He Y., Su J., Cha R. (2023). The Antimicrobial, Hemostatic, and Anti-Adhesion Effects of a Peptide Hydrogel Constructed by the All-d-Enantiomer of Antimicrobial Peptide Jelleine-1. Adv. Healthc. Mater..

[97] Kim D., Jang J., Jin S., Lee J., Jana B., Ryu J.H. (2026). Dual Enzyme-Responsive Zwitterionic Peptide for High Cancer Selectivity via Intralysosomal Self-Assembly. Biomacromolecules.

[98] Fan L., Shen F., Wu D., Ren T., Jiang W. (2024). KGRT peptide incorporated hydrogel with antibacterial activity for wound healing by optimizing cellular functions via ERK/eNOS signaling. Int. J. Biol. Macromol..

[99] Quazi M.Z., Park N. (2023). DNA Hydrogel-Based Nanocomplexes with Cancer-Targeted Delivery and Light-Triggered Peptide Drug Release for Cancer-Specific Therapeutics. Biomacromolecules.

[100] Falcone N., Ermis M., Tamay D.G., Mecwan M., Monirizad M., Mathes T.G., Jucaud V., Choroomi A., de Barros N.R., Zhu Y. (2023). Peptide Hydrogels as Immunomaterials and Their Use in Cancer Immunotherapy Delivery. Adv. Healthc. Mater..

[101] Altunbek M., Gezek M., Buck P., Camci-Unal G. (2024). Development of Human-Derived Photocrosslinkable Gelatin Hydrogels for Tissue Engineering. Biomacromolecules.

[102] Dasgupta S., Sen S., Sathe R.Y., Pophali S., Kadu A., Jain R., Bera S., Roy S., Misra R. (2024). Conformation Controlled Hydrogelation of Minimalistic alpha, gamma Hybrid Peptide. Biomacromolecules.

[103] Goswami A.K., Giduturi V.K., Yerramilli S.N., Chauhan V.S., Yadav N. (2026). Tissue engineering: Hydrogel scaffolds and mechanical properties as key design parameters. Adv. Colloid Interface Sci..

[104] Ciccone G., Azevedo Gonzalez-Oliva M., Versaevel M., Cantini M., Vassalli M., Salmeron-Sanchez M., Gabriele S. (2025). Epithelial Cell Mechanoresponse to Matrix Viscoelasticity and Confinement Within Micropatterned Viscoelastic Hydrogels. Adv. Sci..

[105] Li J., Zhai Y.N., Xu J.P., Zhu X.Y., Yang H.R., Che H.J., Liu C.K., Qu J.B. (2024). An injectable collagen peptide-based hydrogel with desirable antibacterial, self-healing and wound-healing properties based on multiple-dynamic crosslinking. Int. J. Biol. Macromol..

[106] Graeber G., Diaz-Marin C.D., Gaugler L.C., El Fil B. (2024). Intrinsic Water Transport in Moisture-Capturing Hydrogels. Nano Lett..

[107] Huang Y., Zia N., Ma Y., Li T., Walker G.C., Naguib H.E., Kumacheva E. (2024). Colloidal Hydrogel with Staged Sequestration and Release of Molecules Undergoing Competitive Binding. ACS Nano.

[108] Vu H., Peeters E., Hofkens K., Vandemeulebroecke K., T’Sas S., Martin C., Ballet S., Hoogenboom R., Goossens S., Lammens T. (2025). Peptide hydrogels as slow-release formulations of protein therapeutics: Case study of asparaginase-loaded hydrogels. Biomater. Sci..

[109] Elsherbeny A., Bayraktutan H., Gumus N., McCrorie P., Garcia-Sampedro A., Parmar S., Ritchie A.A., Meakin M., Oz U.C., Rahman R. (2025). Pentablock thermoresponsive hydrogels for chemotherapeutic delivery in a pancreatic cancer model. Biomater. Sci..

[110] Yuan L., Zhang Y., Shuai Y., Lei L., Fang Y., Lu K. (2025). Synthesis and bioactivity of pH-response chemically modified antimicrobial peptide hydrogels. Colloids Surf. B Biointerfaces.

[111] Sekar Jeyakumar G.F., Velswamy P., Gunasekaran D., Vincent Paulraj A., Paneerselvam Manimegalai N., Tiruchirappalli Sivagnanam U. (2025). Injectable hybrid hydrogel-mediated calcium-sensing receptor (CaSR) activation for enhanced osteogenesis and bone remodeling. Biomater. Sci..

[112] McCoy A.J., Novick J.S., Zhang I.W., Jew D.L., Putnam A.J. (2025). PEG-Collagen Interpenetrating Networks Support Enhanced Vasculogenic Self-Assembly and Impact Cell-Mediated Remodeling. ACS Biomater. Sci. Eng..

[113] Carvalho E.D., Morais M.R.G., Pego A.P., Barrias C.C., Araujo M. (2024). The interplay between chemical conjugation and biologic performance in the development of alginate-based 3D matrices to mimic neural microenvironments. Carbohydr. Polym..

[114] Park H.J., Kim Y., Lee K.W., Gwon M., Yoon H.C., Yoo T.H. (2023). Coupling hCG-based protease sensors with a commercial pregnancy test strip for simple analyses of protease activities. Biosens. Bioelectron..

[115] Vera-Gonzalez N., Deusenbery C., LaMastro V., Shukla A. (2024). Fungal Enzyme-Responsive Hydrogel Drug Delivery Platform for Triggered Antifungal Release. Adv. Healthc. Mater..

[116] Ding J., Wang T., Lin Z., Li Z., Yang J., Li F., Rong Y., Chen X., He C. (2025). Chiral polypeptide hydrogels regulating local immune microenvironment and anti-tumor immune response. Nat. Commun..

[117] Song H., Su Q., Nie Y., Zhang C., Huang P., Shi S., Liu Q., Wang W. (2023). Supramolecular assembly of a trivalent peptide hydrogel vaccine for cancer immunotherapy. Acta Biomater..

[118] Yang S., Zhu J., Lu C., Chai Y., Cao Z., Lu J., Zhang Z., Zhao H., Huang Y.Y., Yao S. (2022). Aligned fibrin/functionalized self-assembling peptide interpenetrating nanofiber hydrogel presenting multi-cues promotes peripheral nerve functional recovery. Bioact. Mater..

[119] Gray V.P., Amelung C.D., Duti I.J., Laudermilch E.G., Letteri R.A., Lampe K.J. (2022). Biomaterials via peptide assembly: Design, characterization, and application in tissue engineering. Acta Biomater..

[120] Garg S., Jana A., Gupta S., Arshi M.U., Khan J., Gharai P., Roy R., Ghosh S. (2025). Engineered Neuro-Regenerative Peptide Hydrogel for Directed Neural Lineage Reprograming and Regeneration of Sciatic Nerve Injury. Adv. Healthc. Mater..

[121] Hao Z., Feng Q., Wang Y., Wang Y., Li H., Hu Y., Chen T., Wang J., Chen R., Lv X. (2024). A parathyroid hormone related supramolecular peptide for multi-functionalized osteoregeneration. Bioact. Mater..

[122] Bayon-Fernandez A., Mendez-Ardoy A., Alvarez-Lorenzo C., Granja J.R., Montenegro J. (2023). Self-healing cyclic peptide hydrogels. J. Mater. Chem. B.

[123] Hill S.K., England R.M., Perrier S. (2024). Modular design of cyclic peptide—Polymer conjugate nanotubes for delivery and tunable release of anti-cancer drug compounds. J. Control. Release.

[124] Zhang M., Wang H., Dai G.C., Lu P.P., Gao Y.C., Cao M.M., Li Y.J., Rui Y.F. (2024). Injectable self-assembled GDF5-containing dipeptide hydrogels for enhanced tendon repair. Mater. Today Bio.

[125] Dudaryeva O.Y., Cousin L., Krajnovic L., Grobli G., Sapkota V., Ritter L., Deshmukh D., Cui Y., Style R.W., Levato R. (2025). Tunable Bicontinuous Macroporous Cell Culture Scaffolds via Kinetically Controlled Phase Separation. Adv. Mater..

[126] Wang W., Tehoungue A.M., Gao Z., Wang Y., Sun M., Lu Y., Zhang G., Zhang Y. (2026). A pH-responsive multifunctional sericin-pyrrole hydrogel with inherent antibacterial and antioxidant activities. J. Mater. Chem. B.

[127] Yu X., Liu S., Wan Z., Yang X. (2025). Bioactive herbal supramolecular hydrogels with a hierarchical nanofibrillar structure via metal ion mediated co-assembly. Nanoscale.

[128] Li Z., Xing X., Zhao C., Wu Q., Liu J., Qiu X., Wang L. (2024). A rapid interactive chitosan-based medium with antioxidant and pro-vascularization properties for infected burn wound healing. Carbohydr. Polym..

[129] Shi R., Zhan H., Jiang S., Lin K., Yuan C. (2025). DNA-Based Hydrogels for Musculoskeletal Reconstruction: Harnessing Dynamic Programmability and Multimodal Therapeutic Integration. Adv. Sci..

[130] Liu Q., Wu J., Bei H.P., Chen Y., Zhang Y., Peng X., Chen F., Zhao X., Chen Z. (2025). RADA16 and SAAP148 Peptide-Modified Collagen Self-Assembled Hydrogels for Accelerated Healing of Infected Wounds. Adv. Sci..

[131] Zhang W., Liu S., Wang L., Li B., Xie M., Deng Y., Zhang J., Zeng H., Qiu L., Huang L. (2024). Triple-crosslinked double-network alginate/dextran/dendrimer hydrogel with tunable mechanical and adhesive properties: A potential candidate for sutureless keratoplasty. Carbohydr. Polym..

[132] Zhao X., Huang Y., Li Z., Chen J., Luo J., Bai L., Huang H., Cao E., Yin Z., Han Y. (2024). Injectable Self-Expanding/Self-Propelling Hydrogel Adhesive with Procoagulant Activity and Rapid Gelation for Lethal Massive Hemorrhage Management. Adv. Mater..

[133] Yin Y., Liu W., Li L., Cao W., Chen J., Zhao L., Sun X., Duan X., Ren G. (2024). Microwave freeze-drying characteristics and crosslinking behavior of wheat starch-laurel acid complex. Int. J. Biol. Macromol..

[134] McFetridge M.L., Kulkarni K., Hilsenstein V., Del Borgo M.P., Aguilar M.I., Ricardo S.D. (2023). A comparison of fixation methods for SEM analysis of self-assembling peptide hydrogel nanoarchitecture. Nanoscale.

[135] Yu P., Ma Y., Zhu Y., Pei J., Zheng G., Liu Y., Fu K., Cai D., Khattab T., Zhou Y. (2024). Transforming growth factor-beta1-loaded RADA-16 hydrogel scaffold for effective cartilage regeneration. Colloids Surf. B Biointerfaces.

[136] Lu Y., Jin Z., Jian Y., Kong D., Zhou H., Xu Y., Cao R., Xia Z., Yang F., Wu Q. (2025). Metal-hydrogel chelation interfaces for ultrasoft and bidirectional bioelectronics. Natl. Sci. Rev..

[137] Li G., Luo Y., Hu Z., Shi Z., Cao X., Xu R., Mi Y., Yao Y., Mao H., Zhang H. (2025). Recent Advances in Peptide-Functionalized Hydrogels for Bone Tissue Engineering. ACS Biomater. Sci. Eng..

[138] Xie C., Chen Y., Wang L., Liao K., Xue B., Han Y., Li L., Jiang Q. (2024). Recent research of peptide-based hydrogel in nervous regeneration. Bioact. Mater..

[139] Xie X., Li Z., Yang X., Yang B., Zong Z., Wang X., Duan L., Lin S., Li G., Bian L. (2023). Biomimetic Nanofibrillar Hydrogel with Cell-Adaptable Network for Enhancing Cellular Mechanotransduction, Metabolic Energetics, and Bone Regeneration. J. Am. Chem. Soc..

[140] Lv Y., Liang L., Qin M., Jiang R.P., Zong F.F., Wu X., Wu K.L., Liang L. (2025). RGD peptide hydrogel downregulates mechanosignal YAP to inhibit postoperative scarring. Acta Biomater..

[141] Li Z., Sun H., Yin Z., Shi X., Zhao R., Wang W., Zhu Y. (2025). Electrospun Scaffold Co-Modified with YIGSR Peptide and Heparin for Enhanced Skin Wound Healing. Adv. Healthc. Mater..

[142] Ostaszewska A., Michalska Z., Dzierzynska M., Fularczyk M., Bielak K., Morawska A., Kosmala M., Kulig I., Morytz J., Trusiak H. (2025). Beneficial but diverse influence of custom-designed hydrogels modified with IL-4 and SDF-1 peptides on selected populations of cells essential for skeletal muscle regeneration. Int. J. Biol. Macromol..

[143] Liu Y., Li L., He M., Xu Y., Wu Z., Xu X., Luo K., Lv H. (2024). Self-assembled peptide hydrogel loaded with functional peptide Dentonin accelerates vascularized bone tissue regeneration in critical-size bone defects. Regen. Biomater..

[144] Liu G., Guo Q., Liu C., Bai J., Wang H., Li J., Liu D., Yu Q., Shi J., Liu C. (2023). Cytomodulin-10 modified GelMA hydrogel with kartogenin for in-situ osteochondral regeneration. Acta Biomater..

[145] Liang Z., Xiao Q., Wu Y., Song D., Li Y., Chen J., Sun Q., Yang Z., Peng T., Wang Y. (2025). Multidimensional biomimetic strategy integrating structural, functional and biological cues for enhanced tendon-to-bone interface regeneration. Mater. Today Bio.

[146] Oliverio R., Liberelle B., Patenaude V., Moreau V., Thomas E., Virgilio N., Banquy X., De Crescenzo G. (2024). Cofunctionalization of Macroporous Dextran Hydrogels with Adhesive Peptides and Growth Factors Enables Vascular Spheroid Sprouting. ACS Biomater. Sci. Eng..

[147] Jin Z., Huang Z., Huang L., He H., Lan N., Huang C., Liu S., Wei Q., Zheng L., Qin Z. (2026). 3D-bioprinted gum karaya hydrogels with photocurable and stem cell recruitment properties for accelerating cartilage regeneration. Int. J. Biol. Macromol..

[148] Bhandary R., Sen S., Mohanty S., Roy S. (2026). Designing highly tunable laminin-inspired bioactive peptide hydrogel-based biomaterials for directing cellular response. J. Mater. Chem. B.

[149] Zhang M., Yu T., Li J., Yan H., Lyu L., Yu Y., Yang G., Zhang T., Zhou Y., Wang X. (2024). Matrix Metalloproteinase-Responsive Hydrogel with On-Demand Release of Phosphatidylserine Promotes Bone Regeneration Through Immunomodulation. Adv. Sci..

[150] Zhou X., Guo Y., Gao Z., Lv G., Wang X., Zhang M., Zhou Y. (2025). Biodegradable and anti-swelling peptide-based supermolecule hydrogel for eliminating ROS and inhibiting inflammation in acute spinal cord injury repair. Acta Biomater..

[151] Kjar A., Haschert M.R., Zepeda J.C., Simmons A.J., Yates A., Chavarria D., Fernandez M., Robertson G., Abdulrahman A.M., Kim H. (2024). Biofunctionalized gelatin hydrogels support development and maturation of iPSC-derived cortical organoids. Cell Rep..

[152] Wang P., You J., Liu G., Wang Q., Zhang L., Lu X., Qin J., Dong Z., Yi B., Huang Q. (2025). The Combination of Aligned PDA-Fe@PLCL Conduit with Aligned GelMA Hydrogel Promotes Peripheral Nerve Regeneration. Adv. Healthc. Mater..

[153] Gao W., Tang C., Hang D., Chen H., Li D., Yan Y., Wang W., Peng H., Wang X., Zhang E. (2025). Enhancement of spinal cord injury repair in rats using photo-crosslinked GelMA hydrogel combined with mitochondrial transplantation. Bioeng. Transl. Med..

[154] Chen Y., Zhang Q., Yang S., Li G., Shi C., Hu X., Asahina S., Asano N., Zhang Y. (2024). Formulate Adaptive Biphasic Scaffold via Sequential Protein-Instructed Peptide Co-Assembly. Adv. Sci..

[155] Wan L., Liu F., Wang A., He Y., Pan J., Liu Y., Xu J., Xu C., Wu F., Ye Q. (2025). PI3K/Akt pathway-mediated enhancement of bone and vascular regeneration by gelatin/hyaluronic acid/exosome composite scaffold in bone tissue engineering. Biomater. Adv..

[156] Zhang X., Wang S., Yang Q., Chen A., Dong S., Liao L., Zhang C. (2025). 3D Printed Microsphere-Hydrogel Scaffold Facilitates Restoration of Reinnervation in Bone Regeneration through Programmable Release of NGF/BMP-2 Mimetic Peptides. Adv. Healthc. Mater..

[157] Zhang F., Lv M., Wang S., Li M., Wang Y., Hu C., Hu W., Wang X., Wang X., Liu Z. (2024). Ultrasound-triggered biomimetic ultrashort peptide nanofiber hydrogels promote bone regeneration by modulating macrophage and the osteogenic immune microenvironment. Bioact. Mater..

[158] Xue Y., Li J., Jiang T., Han Q., Jing Y., Bai S., Yan X. (2024). Biomimetic Conductive Hydrogel Scaffolds with Anisotropy and Electrical Stimulation for In Vivo Skeletal Muscle Reconstruction. Adv. Healthc. Mater..

[159] Alheib O., da Silva L.P., da Silva Morais A., Mesquita K.A., Pirraco R.P., Reis R.L., Correlo V.M. (2022). Injectable laminin-biofunctionalized gellan gum hydrogels loaded with myoblasts for skeletal muscle regeneration. Acta Biomater..

[160] Li Y., Liu S., Zhang J., Wang Y., Lu H., Zhang Y., Song G., Niu F., Shen Y., Midgley A.C. (2024). Elastic porous microspheres/extracellular matrix hydrogel injectable composites releasing dual bio-factors enable tissue regeneration. Nat. Commun..

[161] Griveau L., Bouvet M., Christin E., Paret C., Lecoq L., Radix S., Laumonier T., Sohier J., Gache V. (2024). Synthetic injectable and porous hydrogels for the formation of skeletal muscle fibers: Novel perspectives for the acellular repair of substantial volumetric muscle loss. J. Tissue Eng..

[162] Li R., Yang R., Zhang Y., Yao S., Xu Y., Yu P., Zhuang Y., Cui W., Wang L. (2024). Graded Modulation of Inflammation by Metal Ion-Coordinated Peptide-Based Hydrogel Chemical Regulators Promotes Tendon-Bone Junction Regeneration. ACS Appl. Mater. Interfaces.

[163] Huang Z., Cheng J., Su W. (2023). A Double Cross-Linked Injectable Hydrogel Derived from Muscular Decellularized Matrix Promotes Myoblast Proliferation and Myogenic Differentiation. Materials.

[164] Zhu D., Hu Y., Kong X., Luo Y., Zhang Y., Wu Y., Tan J., Chen J., Xu T., Zhu L. (2024). Enhanced burn wound healing by controlled-release 3D ADMSC-derived exosome-loaded hyaluronan hydrogel. Regen. Biomater..

[165] Yang K., Yang J., Chen R., Dong Q., Zhou Y. (2024). Fast Self-Healing Hyaluronic Acid Hydrogel with a Double-Dynamic Network for Skin Wound Repair. ACS Appl. Mater. Interfaces.

[166] Jiang S., Zheng Y., Yang P., Liu H., Zhang W., Jiang Y., Yan L., Weng J., Lin F., Sun H. (2026). Polyphenol-enhanced extreme-environment adaptive hydrogels for high-altitude burn wound repair. J. Mater. Chem. B.

[167] Wei H., Zhang H., Yan S., Hou K., Chen R., Cao R., Zhu M. (2025). 3D-Printed ROS-Scavenging, Proangiogenic, and Biodegradable Hydrogel for Enhancing Burn Wound Healing. ACS Appl. Mater. Interfaces.

[168] Miao X., Davoudi M., Sadegh-Nejadi S., Ghahari S.A., Bagherieh M., Afrisham R. (2025). Skin regenerative potential of hydrogel matrices incorporated with stem cell-derived extracellular vesicles enriched with MicroRNAs: A systematic review. Mol. Cell Biochem..

[169] Liu Y., Okesola B.O., Osuna de la Pena D., Li W., Lin M.L., Trabulo S., Tatari M., Lawlor R.T., Scarpa A., Wang W. (2024). A Self-Assembled 3D Model Demonstrates How Stiffness Educates Tumor Cell Phenotypes and Therapy Resistance in Pancreatic Cancer. Adv. Healthc. Mater..

[170] Kopyeva I., Bretherton R.C., Ayers J.L., Yu M., Grady W.M., DeForest C.A. (2025). Matrix Stiffness and Biochemistry Govern Colorectal Cancer Cell Growth and Signaling in User-Programmable Synthetic Hydrogels. ACS Biomater. Sci. Eng..

[171] Lopes M., Torrado M., Barth D., Santos S.D., Sever-Bahcekapili M., Tekinay A.B., Guler M.O., Cleymand F., Pego A.P., Borges J. (2023). Supramolecular presentation of bioinstructive peptides on soft multilayered nanobiomaterials stimulates neurite outgrowth. Biomater. Sci..

[172] Sung T.C., Chen Y.H., Wang T., Qian L., Chao W.H., Liu J., Pang J., Ling Q.D., Lee H.H., Higuchi A. (2024). Design of dual peptide-conjugated hydrogels for proliferation and differentiation of human pluripotent stem cells. Mater. Today Bio.

[173] Nunomura S., Nanri Y., Honda Y., Takedomi H., Okada T., Tun X., Conway S.J., Kitajima I., Ebihara N., Matsuda A. (2026). Periostin promotes inflammation and neovascularization in atopic keratoconjunctivitis. J. Allergy Clin. Immunol..

[174] Pu M., Cao H., Zhang H., Wang T., Li Y., Xiao S., Gu Z. (2024). ROS-responsive hydrogels: From design and additive manufacturing to biomedical applications. Mater. Horiz..

[175] Kang H., Liu X., Ge D., Zeng Y. (2025). Revolutionizing bladder cancer research: Harnessing 3D organoid technology to decode tumor heterogeneity and propel personalized therapeutics. Biochim. Biophys. Acta Rev. Cancer.

[176] Lee A.L.Z., Balakrishnan N., Ng J.Y., Liu S., Ong Z.Y., Wang Y., Gao S., Yang Y.Y. (2024). Injectable Hydrogels Prepared Using Novel Synthetic Short Peptides with Defined Structure and Gelatin as Scaffolds to Support Cell and Tissue Growth. Adv. Healthc. Mater..

[177] Jergitsch M., Perez-Amodio S., Delgado L.M., Perez R.A., Mateos-Timoneda M.A. (2026). 3D coaxial bioprinting of RADA16-I self-assembling peptide hydrogel. Mater. Today Bio.

[178] Rothe R., Xu Y., Wodtke J., Brandt F., Meister S., Laube M., Lollini P.L., Zhang Y., Pietzsch J., Hauser S. (2024). Programmable Release of Chemotherapeutics from Ferrocene-Based Injectable Hydrogels Slows Melanoma Growth. Adv. Healthc. Mater..

[179] Chen Z., Wu H., Wang Y., Rao Y., Yan J., Ran B., Zeng Q., Yang X., Cao J., Cao H. (2024). Enhancing melanoma therapy by modulating the immunosuppressive microenvironment with an MMP-2 sensitive and nHA/GNE co-encapsulated hydrogel. Acta Biomater..

[180] Wang K., Zhang J., Liu W., Kong L., Li H., Zhou L., Zhang J., Hu X., Qiu X., Wang X. (2026). Bioresponsive Dual Microspheres Embedded in a Hydrogel: A Sequentially Triggered Strategy for Regulating Diabetic Wound Healing. Adv. Healthc. Mater..

[181] Liu Q., Xie J., Zhou R., Deng J., Nie W., Sun S., Wang H., Shi C. (2025). A matrix metalloproteinase-responsive hydrogel system controls angiogenic peptide release for repair of cerebral ischemia/reperfusion injury. Neural Regen. Res..

[182] Cauwenbergh T., Ballet S., Martin C. (2025). Peptide hydrogel-drug conjugates for tailored disease treatment. Mater. Today Bio.

[183] Du L., Wu Y., Liu Q., Xu Y., Dai W., Li X., Lei L., Bao Z. (2025). Bioactive peptide based supramolecular hydrogel as an efficient drug/cell delivery platform for ophthalmic application. Int. J. Pharm..

[184] Cai X., Zhang M., Zou J., Wang L., Zhan Y., Li D., Jiang T., Alim N., Liu Z., Yang J. (2024). A novel self-assembling peptide nanofiber hydrogel with glucagon-like peptide-1 functionality enhances islet survival to improve islet transplantation outcome in diabetes treatment. J. Nanobiotechnology.

[185] Liu J., Xi Z., Fan C., Mei Y., Zhao J., Jiang Y., Zhao M., Xu L. (2024). Hydrogels for Nucleic Acid Drugs Delivery. Adv. Healthc. Mater..

[186] Chen Y.K., Kulkarni K., Aguilar M.I., Broughton B.R.S., Del Borgo M.P. (2026). An antagomiR-loaded beta-peptide hydrogel promotes functional recovery in mice post-ischaemic stroke. Biomater. Adv..

[187] Zhang X., Zhang Y., Rong X., Tang C., Liu H., Yue L., Su R., Wang Y., Qi W. (2024). Alkylated RALA-Derived Peptides for Efficient Gene Delivery. Biomacromolecules.

[188] He C., Wang Y., Fang X., Jiang W., Liu S., Yi X., Zhang K., Lin H., Zeng Q., Zhu X. (2025). Charge microenvironment and bioactivity of in situ-formed PEG-RGD dual hydrogel dressings promote wound healing. J. Mater. Chem. B.

[189] Chen X., Xu Z., Gao Y., Chen Y., Yin W., Liu Z., Cui W., Li Y., Sun J., Yang Y. (2024). Framework Nucleic Acid-Based Selective Cell Catcher for Endogenous Stem Cell Recruitment. Adv. Mater..

[190] Lv K., Lou P., Liu S., Wang Y., Yang J., Zhou P., Zhou X., Lu Y., Wang H., Cheng J. (2024). Injectable Multifunctional Composite Hydrogel as a Combination Therapy for Preventing Postsurgical Adhesion. Small.

[191] Kim Y.H., Kim S., Ju H.J., Han M.J., Park Y., Kim E., Choi H.S., Choi S., Kim M.S. (2023). In-situ wound healing by SDF-1-mimic peptide-loaded click crosslinked hyaluronic acid scaffold. J. Control. Release.

[192] Sun Y., Yao X., Zhang Y., Zhang W., Zhu C., Shen C., Wang Y., Wang X. (2025). Zinc Oxide-Copper Sulfide Nanozyme Hydrogels for Bone Defect Repair by Modulating the Bone Immune Microenvironment and Promoting Osteogenesis/Angiogenesis. ACS Appl. Mater. Interfaces.

[193] Cai X., Xie Q., Zhao G., Linghu X., Huang W., Xiao C., Song W., Xu N., Zhou J., Xu W. (2025). Minimally Invasive Injection of Magnesium-Doped Bioactive Glass Hydrogels for Immunomodulatory Repair of Bone Defects. ACS Biomater. Sci. Eng..

[194] Mai Y., Wang H., Lu J., Shi S., Cai Y., Zhang W., Xie S., Huang R., Ji S., Qu X. (2025). Catalyst-modulated hydrogel dynamics for decoupling viscoelasticity and directing macrophage fate for diabetic wound healing. Bioact. Mater..

[195] Zhang T., Shao M., Li H., Chen X., Zhang R., Wu J., Wang J., Guo Y. (2024). Decellularized Amnion Membrane Triggers Macrophage Polarization for Desired Host Immune Response. Adv. Healthc. Mater..

[196] Liang L., Xu W., Shen A., Fu X., Cen H., Wang S., Lin Z., Zhang L., Lin F., Zhang X. (2023). Inhibition of YAP1 activity ameliorates acute lung injury through promotion of M2 macrophage polarization. MedComm.

[197] Kong N., Chen D., Liang J., Wu B., Wang H. (2024). Reprogramming Macrophages toward Pro-inflammatory Polarization by Peptide Hydrogel. Biomacromolecules.

[198] Yang X., Liu Z., Zhou J., Guo J., Han T., Liu Y., Li Y., Bai Y., Xing Y., Wu J. (2024). SPP1 promotes the polarization of M2 macrophages through the Jak2/Stat3 signaling pathway and accelerates the progression of idiopathic pulmonary fibrosis. Int. J. Mol. Med..

[199] Zhao T., Zhong G., Wang Y., Cao R., Song S., Li Y., Wan G., Sun H., Huang M., Bi H. (2024). Pregnane X Receptor Activation in Liver Macrophages Protects against Endotoxin-Induced Liver Injury. Adv. Sci..

[200] Zhen J., Bai J., Liu J., Men H., Yu H. (2024). Ginsenoside RG1-induced mesenchymal stem cells alleviate diabetic cardiomyopathy through secreting exosomal circNOTCH1 to promote macrophage M2 polarization. Phytother. Res..

[201] Ji J., He Q., Xia Y., Sha X., Liang Q., Xu Y., Chen P., Dong C., Zhao R., Yang J. (2024). Circulating plasma derived exosomes from systemic lupus erythematosus aggravate lupus nephritis through miR-122-5p/FOXO3-mediated macrophage activation. J. Nanobiotechnology.

[202] Pineda-Hernandez A., Castilla-Casadiego D.A., Morton L.D., Giordano-Nguyen S.A., Halwachs K.N., Rosales A.M. (2025). Tunable hydrogel networks by varying secondary structures of hydrophilic peptoids provide viable 3D cell culture platforms for hMSCs. Biomater. Sci..

[203] Lv X., Niu W., Zhang B., Chen J., Yang S., Xue Y., Dong Y., Yuan P., Pan Y., Tan J. (2024). Self-Assembled Peptide Hydrogels Loaded with Umbilical Cord-Derived Mesenchymal Stem Cells Repairing Injured Endometrium and Restoring Fertility. Adv. Healthc. Mater..

[204] Ji W., Wen J., Tong N., Guo F., Zheng J., Wen X., Liu J., Zhang N., Hou B. (2026). A pH-responsive hyaluronic acid hydrogel facilitates lesion-localized integrin alpha5beta1 agonism to attenuate osteopontin signaling and fibrosis in intrauterine adhesions. Acta Biomater..

[205] Wu Q., Jia K., Liu Y., Zhang Y., Jiang N., Li L., Liu Y., Li L. (2026). The effect of the physicochemical properties of hydrogels on chronic inflammation by macrophage polarization. Biomater. Sci..

[206] Wang L., Zhang H., Li Y., Li L. (2024). TPX2 influences the regulation of macrophage polarization via the NF-kappaB pathway in lung adenocarcinoma. Life Sci..

[207] Chen J., Cui M., He L., Mu Y., Hu N., Guan X. (2023). Engineered elastin-like polypeptide-based hydrogel delivering chemotherapeutics and PD-L1 antibodies for potentiated cancer immunotherapy. J. Mater. Chem. B.

[208] Ji P., Sun W., Zhang S., Xing Y., Wang C., Wei M., Li Q., Ji G., Yang G. (2023). Modular Hydrogel Vaccine for Programmable and Coordinate Elicitation of Cancer Immunotherapy. Adv. Sci..

[209] Wang F., Zeng Y., Yan M., Zhou Z., Feng L., Yang F., Zhao W., Hu Y. (2026). Self-transforming hydrogel mimicking tertiary lymph nodes to activate cGAS-STING pathway for enhanced antitumor immunotherapy. Sci. Adv..

[210] Wu B., Liang J., Yang X., Fang Y., Kong N., Chen D., Wang H. (2024). A Programmable Peptidic Hydrogel Adjuvant for Personalized Immunotherapy in Resected Stage Tumors. J. Am. Chem. Soc..

[211] Zheng W., Wang Y., Sun H., Bao S., Ge S., Quan C. (2025). The role of Fusobacterium nucleatum in macrophage M2 polarization and NF-kappaB pathway activation in colorectal cancer. Front. Immunol..

[212] Nie R., Zhang Q.Y., Feng Z.Y., Huang K., Zou C.Y., Fan M.H., Zhang Y.Q., Zhang J.Y., Li-Ling J., Tan B. (2024). Hydrogel-based immunoregulation of macrophages for tissue repair and regeneration. Int. J. Biol. Macromol..

[213] Lai C.M., Chen W.J., Qin Y., Xu D., Lai Y.K., He S.H. (2025). Innovative Hydrogel Design: Tailoring Immunomodulation for Optimal Chronic Wound Recovery. Adv. Sci..

[214] Feng J., Wang Z., Li X., Bao C., Xiao Y. (2025). Facile Formulation of a Resveratrol-Mediated Multibond Network Hydrogel with Efficient Sustainable Antibacterial, Reactive Oxygen Species Scavenging, Pro-Angiogenesis, and Immunomodulation Activities for Accelerating Infected Wound Healing. ACS Appl. Mater. Interfaces.

[215] Yao J., Tian H., Meng Y., Wang J., Feng J., Ba Q., Kong Y., Xiao S., Gong W., Wang Y. (2025). A Novel Gelatinase-Responsive Self-Assembled Antimicrobial Peptide for Combating Drug-Resistant Bacterial Infection. ACS Appl. Mater. Interfaces.

[216] Shen Z., Guo Z., Zhou L., Wang Y., Zhang J., Hu J., Zhang Y. (2020). Biomembrane induced in situ self-assembly of peptide with enhanced antimicrobial activity. Biomater. Sci..

[217] Rathee G., Puertas-Segura A., Blair J., Tzanov T. (2025). From lab to pilot: Sonochemical coating of PDDA-based polymer dots for scalable, wash-durable antibacterial textiles. Ultrason. Sonochem..

[218] Chen Z., Li C., Wang L., Luo Y., Yang Y., Han Q., Zhang J., Shi Y., Sun Y., Song Y. (2026). A WR3-NH(2)-loaded polysaccharide hydrogel with antibacterial, anti-inflammatory, and pro-healing properties for enhanced wound healing. Mater. Today Bio.

[219] Wang Y., Shi J., Wang M., Zhang L., Wang R., Zhang J., Qing H., Duan J., Zhang X., Pu G. (2024). pH-Responsive Co-Assembled Peptide Hydrogel to Inhibit Drug-Resistant Bacterial Infection and Promote Wound Healing. ACS Appl. Mater. Interfaces.

[220] Gao F., Ahmed A., Cong H., Yu B., Shen Y. (2023). Effective Strategies for Developing Potent, Broad-Spectrum Antibacterial and Wound Healing Promotion from Short-Chain Antimicrobial Peptides. ACS Appl. Mater. Interfaces.

[221] Cai D., Li C., Zhu T., Li R., Zhang M., Li X., Liu Y., Dai Z., Wan L., Lu H. (2025). Antimicrobial peptide hydrogels: Synthesis, ROS regulation mechanism, and multimodal therapeutic applications in drug delivery systems. J. Mater. Chem. B.

[222] Hao R., Ye X., Chen X., Du J., Tian F., Zhang L., Ma G., Rao F., Xue J. (2024). Integrating Bioactive Graded Hydrogel with Radially Aligned Nanofibers to Dynamically Manipulate Wound Healing Process. ACS Appl. Mater. Interfaces.

[223] Lu Y., Kang W., Yu Y., Lu H., Wang Y., Xu Z., Zeng J., Qin M., Xu X. (2024). A synergistically antimicrobial and antioxidant hyaluronic acid hydrogel for infected wounds. Int. J. Biol. Macromol..

[224] Petit N., Gomes A., Chang Y.J., Da Silva J., Leal E.C., Carvalho E., Gomes P., Browne S. (2025). Development of a bioactive hyaluronic acid hydrogel functionalised with antimicrobial peptides for the treatment of chronic wounds. Biomater. Sci..

[225] Guo Y., Gao F., Rafiq M., Yu B., Cong H., Shen Y. (2024). Preparation of antimicrobial peptides and their combination with hydrogels for wound healing applications. Int. J. Biol. Macromol..

[226] Lin X., Fu T., Lei Y., Xu J., Wang S., He F., Xie Z., Zhang L. (2023). An injectable and light curable hyaluronic acid composite gel with anti-biofilm, anti-inflammatory and pro-healing characteristics for accelerating infected wound healing. Int. J. Biol. Macromol..

[227] Ni L., Zhang X., Gao J., Yue J., Zhang L., Pan G. (2025). Dual peptide-coordinated dynamic hydrogel with antibacterial and proangiogenic activities for infected skin wounds. Int. J. Biol. Macromol..

[228] Ding K., Liao M., Wang Y., Lu J.R. (2025). Advances in Composite Stimuli-Responsive Hydrogels for Wound Healing: Mechanisms and Applications. Gels.

[229] Tang Z., Deng L., Zhang J., Jiang T., Xiang H., Chen Y., Liu H., Cai Z., Cui W., Xiong Y. (2024). Intelligent Hydrogel-Assisted Hepatocellular Carcinoma Therapy. Research.

[230] Peng Y., Liu H., Liang X., Cao L., Teng M., Chen H., Li Z., Peng X., Mao J., Cheng H. (2025). Self-assembling chemodrug fiber-hydrogel for transarterial chemoembolization and radiotherapy-enhanced antitumor immunity. J. Control. Release.

[231] Zhao Y., Gong J., Liu H., Huang H., Tan W.S., Cai H. (2024). A chemically defined, mechanically tunable, and bioactive hyaluronic acid/alginate double-network hydrogel for liver cancer organoid construction. Int. J. Biol. Macromol..

[232] Choi Y.M., Kim D.H., Jang J., Kim B.J. (2023). A hepatitis B virus-derived peptide combined with HBsAg exerts an anti-HBV effect in an HBV transgenic mouse model as a therapeutic vaccine. Front. Immunol..

[233] Lin C., Luo L., Xun Z., Zhu C., Huang Y., Ye Y., Zhang J., Chen T., Wu S., Zhan F. (2024). Novel function of MOTS-c in mitochondrial remodelling contributes to its antiviral role during HBV infection. Gut.

[234] Chen Z., Zhao G., Qu Y., Tang Q., Yang S., Zhou C., Li M., Kang Y., Tan P., Ma X. (2025). Structure- or size-transformable peptide-based antibacterial biomaterials: Design strategies, functions, and applications. Acta Biomater..

[235] Guilbaud-Chereau C., Dinesh B., Wagner L., Chaloin O., Menard-Moyon C., Bianco A. (2022). Aromatic Dipeptide Homologue-Based Hydrogels for Photocontrolled Drug Release. Nanomaterials.

[236] Ovrebo O., Perale G., Wojciechowski J.P., Echalier C., Jeffers J.R.T., Stevens M.M., Haugen H.J., Rossi F. (2022). Design and clinical application of injectable hydrogels for musculoskeletal therapy. Bioeng. Transl. Med..

[237] Imtiaz S., Ferdous U.T., Nizela A., Hasan A., Shakoor A., Zia A.W., Uddin S. (2025). Mechanistic study of cancer drug delivery: Current techniques, limitations, and future prospects. Eur. J. Med. Chem..

[238] Adnan S.B., Lim F., Ahmad H., Maarof M., Fauzi M.B., Md Fadilah N.I. (2026). Peptide-Based Nanocarriers for Targeted Drug Delivery: Recent Advances, Strategies, and Therapeutic Frontiers. Int. J. Nanomed..

[239] Robang A.S., Roy A., Dodd O.J., He D., Le J.V., McShan A.C., Hu Y., Kumar V.A., Paravastu A.K. (2024). Structural Consequences of Introducing Bioactive Domains to Designer beta-Sheet Peptide Self-Assemblies. Biomacromolecules.

[240] Xiang G., Yin B., Shiroud Heidari B., Youssef G., Gosecka M., Gosecki M., Torres F.G., Wong S.H.D., Dodda J.M. (2025). Programmable Hydrogels: Frontiers in Dynamic Closed-Loop Systems, Biomimetic Synergy, and Clinical Translation. Adv. Sci..

[241] Zhang P., Hao Y., Xie M., Wang H., Gao J. (2025). Molecular craftsmanship: Reforging peptide backbones for enhanced drug design. Drug Discov. Today.

[242] Wang S., Liu Y., Zhou Y., Zheng W., Mao Y. (2025). Biomimetic peptides in bone tissue engineering: From function to application. Colloids Surf. B Biointerfaces.

[243] Zhou H., Zheng H., Yao W., Sun H., Yang Y.G., Li Z., Song D., Zhang Y., Sun T. (2025). Spatiotemporally controlled delivery of biomacromolecules via injectable hydrogels for precision modulation of the tumor immune microenvironment. J. Nanobiotechnology.

[244] Li J., Xue Y., Wang A., Tian S., Li Q., Bai S. (2022). Polyaniline Functionalized Peptide Self-Assembled Conductive Hydrogel for 3D Cell Culture. Gels.

[245] Das S., Das D. (2021). Rational Design of Peptide-based Smart Hydrogels for Therapeutic Applications. Front. Chem..

[246] Ligorio C., Cianciosi A., Tognato R., Natta M., Parolini R., Ardicli S., Babayev H., Sapjanskaite I., Kilgour S.L., Homer R. (2026). Bioconvergence of sound-guided and supramolecular assembly strategies to create peptide-protein composite hydrogels with predictable shape-to-function features. Mater. Today Bio.

[247] Wang L., Gu X., Zhao Y., Tian J., Ma X., Tong M. (2026). Advances in Molecular Dynamics Simulations for Hydrogels and Nanocomposite-Reinforced Hydrogels: Multiscale Simulation Strategies and Future Directions. Gels.

[248] Vera-Tizatl A.L., van der Hee R., Cornelissen J., Vera-Tizatl C.E., Abayazid M., Futterer J.J. (2024). Liver-tumor mimics as a potential translational framework for planning and testing irreversible electroporation with multiple electrodes. Bioeng. Transl. Med..

[249] Yuan F., Peng D., Lu M., Zhang K., Mi P., Xu J. (2025). Ultrasound-responsive nanocarriers for cancer therapy: Physiochemical features-directed design. J. Control. Release.

[250] Xu T., Wang J., Zhao S., Chen D., Zhang H., Fang Y., Kong N., Zhou Z., Li W., Wang H. (2023). Accelerating the prediction and discovery of peptide hydrogels with human-in-the-loop. Nat. Commun..

[251] Zhang S., Wang H., Liu F., Su Y., Han K., Liu Y., Guan F., Liu H., Ma S. (2025). Artificial intelligence-enabled hydrogels: Innovations and applications. J. Mater. Chem. B.

[252] Li Z., Song P., Li G., Han Y., Ren X., Bai L., Su J. (2024). AI energized hydrogel design, optimization and application in biomedicine. Mater. Today Bio.

[253] Prypoten V., Norton R.S., Chalmers D.K. (2026). Contact Parallel Cascade Selection Molecular Dynamics (cPaCS-MD) for Accurate In Silico Prediction of Peptide Binding Free Energy. J. Chem. Inf. Model..

